# Simulation experiment to test strategies of geomagnetic navigation during long-distance bird migration

**DOI:** 10.1186/s40462-021-00283-5

**Published:** 2021-09-15

**Authors:** Beate Zein, Jed A. Long, Kamran Safi, Andrea Kölzsch, Martin Wikelski, Helmut Kruckenberg, Urška Demšar

**Affiliations:** 1 School of Geography and Sustainable Development, Irvine Building, University of St Andrews, North Street, KY16 9AL St Andrews, Scotland, UK; 2 Department of Geography & Environment, Western University, London, ON Canada; 3 Department of Migration, MPI of Animal Behavior, Radolfzell, Germany; 4 Department of Biology, University of Konstanz, Konstanz, Germany; 5 Centre for the Advanced Study of Collective Behaviour, University of Konstanz, 78457 Konstanz, Germany; 6 Institute for Wetlands and Waterbird Research E.V, Verden (Aller), Germany

**Keywords:** Bird migration, Earth’s magnetic field, Geomagnetic navigation, Greater white-fronted geese, Method development, Navigational strategies, Random walk models

## Abstract

**Background:**

Different theories suggest birds may use compass or map navigational systems associated with Earth’s magnetic intensity or inclination, especially during migratory flights. These theories have only been tested by considering properties of the Earth’s magnetic field at coarse temporal scales, typically ignoring the temporal dynamics of geomagnetic values that may affect migratory navigational capacity.

**Methods:**

We designed a simulation experiment to study if and how birds use the geomagnetic field during migration by using both high resolution GPS tracking data and geomagnetic data at relatively fine spatial and temporal resolutions in comparison to previous studies. Our simulations use correlated random walks (CRW) and correlated random bridge (CRB) models to model different navigational strategies based on underlying dynamic geomagnetic data. We translated navigational strategies associated with geomagnetic cues into probability surfaces that are included in the random walk models. Simulated trajectories from these models were compared to the actual GPS trajectories of migratory birds using 3 different similarity measurements to evaluate which of the strategies was most likely to have occurred.

**Results and conclusion:**

We designed a simulation experiment which can be applied to different wildlife species under varying conditions worldwide. In the case of our example species, we found that a compass-type strategy based on taxis, defined as movement towards an extreme value, produced the closest and most similar trajectories when compared to original GPS tracking data in CRW models. Our results indicate less evidence for map navigation (constant heading and bi-gradient taxis navigation). Additionally, our results indicate a multifactorial navigational mechanism necessitating more than one cue for successful navigation to the target. This is apparent from our simulations because the modelled endpoints of the trajectories of the CRW models do not reach close proximity to the target location of the GPS trajectory when simulated with geomagnetic navigational strategies alone. Additionally, the magnitude of the effect of the geomagnetic cues during navigation in our models was low in our CRB models. More research on the scale effects of the geomagnetic field on navigation, along with temporally varying geomagnetic data could be useful for further improving future models.

**Supplementary Information:**

The online version contains supplementary material available at 10.1186/s40462-021-00283-5.

## Background

Long-distance migratory flights are energetically costly [1, 2] and movements off course can result in fatal outcomes. To be competitive at feeding grounds or breeding sites, it is crucial for most animals to arrive at the correct location at the right time [3]. Therefore, an accurate and adaptable navigational mechanism robust to environmental change is required. In particular, birds are known to use a multifactorial navigational mechanism, especially during migration [4–6] but it is still unclear how different potential methods of navigation interact or function. A highly debated navigational mechanism involves birds using the geomagnetic field and different potential navigational strategies (compass or map) associated with different geomagnetic properties (e.g., intensity, inclination). Research has demonstrated the importance of the geomagnetic field in specific instances, for example when the magnetic compass of songbirds can be experimentally recalibrated during a single migratory night [7] and through evidence of individuals becoming disoriented during local geomagnetic changes [8–10]. Since the geomagnetic field is highly dynamic on a global scale, the level at which birds navigate using the geomagnetic field is of increasing relevance [11].

Birds may use multiple different mechanisms to detect properties of the Earth’s magnetic field. Specifically, some birds have been suggested to possess an iron-based sensory mechanism in the beak which detects magnetic intensities [12, 13]. Others may have a protein-based sensory system in the eye for detecting magnetic inclination [14, 15]. A third potential mechanism is suspected to be located in the ear [16]. The sensitivity for detecting changes in magnetic intensity has been estimated through behavioural experiments to be in the range of 20–50 nanoTesla (nT) [17, 18] and 50-200nT in neurophysiological experiments [19, 20]. Determining the sensory sensitivity of birds is important because the intensity of the Earth’s magnetic field changes on average 10nT over every km with local daily variation of about 30-100nT in mid-latitudes and up to 1000nT in polar regions. The sensitivity in resolving differences has been shown to be in the order of less than 1–5 degrees [21–23] and around 0.009° per km. The scale at which the geomagnetic field varies during a migratory flight of a bird is therefore well within the range of what birds can detect, making use of these cues for navigation highly relevant and plausible.

Geomagnetic fields may be used for two different types of navigational strategies, compass and map. Birds can use the Earth’s magnetic field to derive a constant direction for compass navigation, similar to using a simple magnetic compass to maintain a constant direction [4, 24–26]. This is however affected by local and global changes in the magnetic field and needs to be regularly calibrated [7, 27–29]. Map navigation is a more complex navigational strategy and requires more precise information about the geomagnetic field. It is defined by an individual’s ability to localise themselves in accordance with a desired goal based on a two-dimensional grid [30–34]. While both values of Earth’s magnetic intensity and inclination could be used for compass navigation independently, map navigation would require at least the use of two different cues (e.g., intensity and inclination). Additionally, a combination of map navigation with additional navigational mechanism might be possible [24, 33, 35–38].

There is on-going debate about how the geomagnetic field might be used by birds, and about which navigational strategies are likely [33]. Different approaches to study geomagnetic bird navigation in the past relied on behavioural [10, 17, 18, 21–23, 39], neurophysiological [19, 20], or displacement experiments [40–43]. More recently, scientists have explored different modelling approaches [44] to study navigational strategies during migration. However, a key limitation of such modelling approaches is the quality of the geomagnetic data that are available. Existing approaches have generally been based on static representations of the geomagnetic field, which fails to capture the fact that in reality it is constantly changing and that these local dynamics are within the sensory range of birds.

The rapid growth of GPS tracking and the emergence of new methods and models for collecting better quality data on the geomagnetic field mean that a data-driven approach may yield additional insights about how birds navigate in relation to the geomagnetic field. Agent Based Models (ABMs), for example, can be used to simulate individual movement and simultaneously incorporate interactions with the environment [45–52]. Mathematically the movement path within an ABM can be modelled as a random walk [53], which can be used to generate a movement track, simulated purely from random decisions of the individual—affecting step length and turning angle. Random walk models can be extended in many ways to include for example bias in movement direction based on previous steps (correlated random walks (CRW)) and/or a bias/drift component based on external factors [50].

In this study, we design a simulation experiment to study potential navigational mechanisms using the geomagnetic field built upon high-resolution GPS tracking data and dynamic models of the geomagnetic field. This is a new approach that integrates temporally varying modelled geomagnetic data into random walk models representing different navigational strategies. We build the parameters of our individual-based models using 14 individual autumn migrations of greater white-fronted geese (Anser a. albifrons) captured with GPS-tracking by applying 5 different navigational strategies. Using this simulation environment, we explore the following two questions: (1) which modelled navigational strategy most closely aligns with the real GPS tracking data; and (2) which geomagnetic navigational mechanisms best predict migratory movements.

## Methods

### GPS data

We used GPS tracks of adult greater white-fronted geese that migrated between their Russian Arctic breeding grounds and West-European wintering sites. These geese are a good model species as they migrate at day time and night time [54], making it more likely that they use multiple navigational mechanisms including geomagnetic navigation. The geese were caught either in family groups during moult on Kolguev Island (Russia) or during winter in the Netherlands or Northern Germany and equipped with high-resolution GPS neckband tags (madebytheo, 35 g; see details in Kölzsch et al., 2016) [54]. We only used data from autumn migration (September–November), as the geese stop very little then, [54] and only included animals which completed the whole migration in 3 days or less, making it easier to model constant flight trajectories. The solar tags were programmed to collect data at a temporal resolution of one position every 30 min, however due to low recharge in autumn resolutions differed. We selected only one animal out of each flock to avoid the impact of it being a social bird, as one individual would be representative of the migratory decisions of the entire flock [54].

**Fig. 1:**
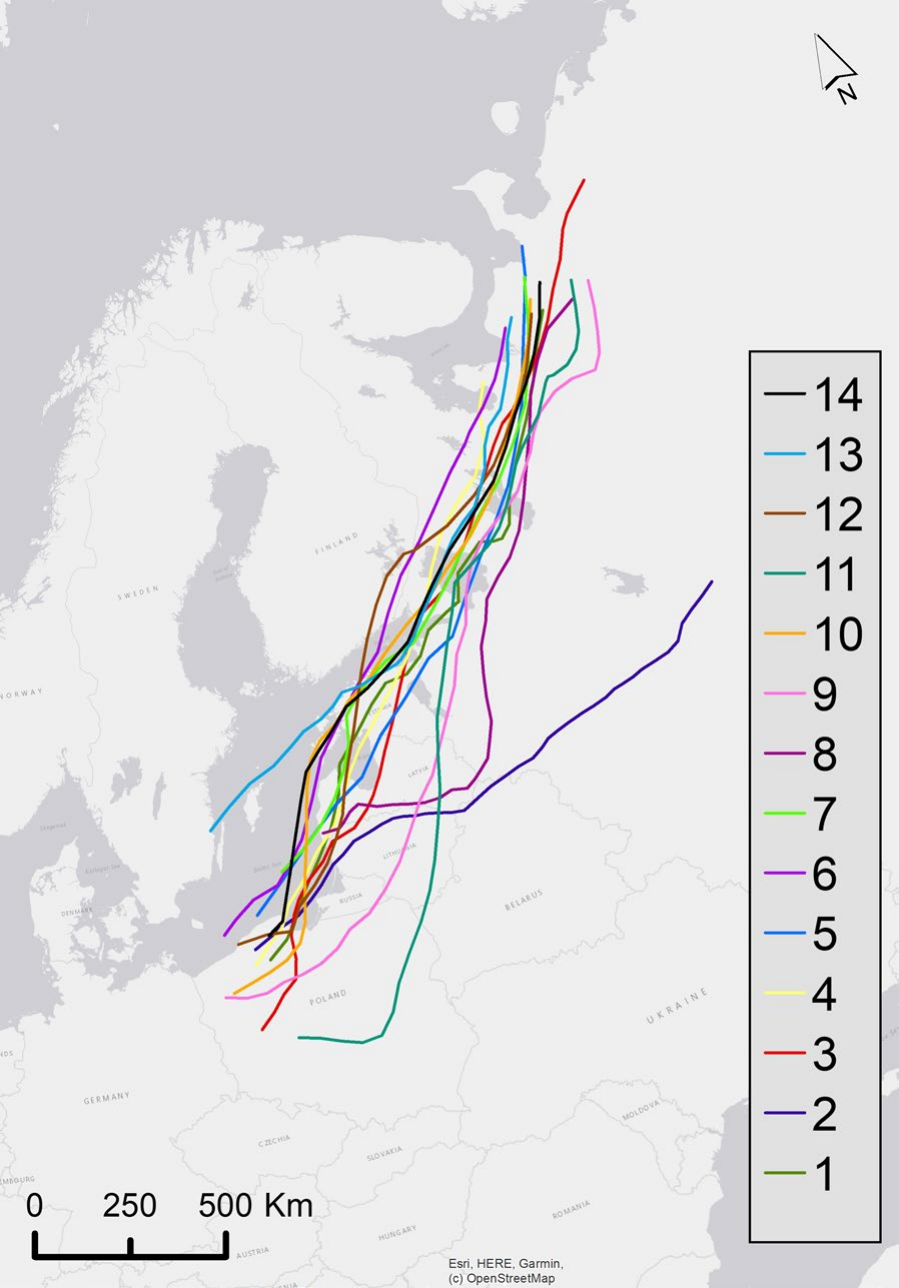
Autumn migratory trajectories of individual greater white-fronted geese from 2016 to 2019

We pre-processed the GPS data to remove outliers. Our focus was only on migratory flights, so we extracted data from migratory periods, excluding data associated with summer and wintering habitat areas. To exclude the data from the summer locations we generated a 500 km exclusion zone around the northernmost point of each bird in each year which was based on the maximum distance travelled in the breeding location [55]. Greater white-fronted geese are less localised in wintering habitats and therefore we excluded data from the winter locations with a 700 km exclusion zone around the southernmost and westernmost points for each bird in each year. We only used bird trajectories where the length of the migratory track was more than 1500 km. Since birds might stop along a migratory flight, only points with ground speeds higher than 6 km/h can be assumed as flying. For example, a study on bar-headed and barnacle geese found maximum ground speed was 1.17 m/s = 4.2 km/h [56]. We removed all points associated with speeds less than 6 km/h to exclude stopover locations from the migratory flight. In total, we analysed 14 autumn migrations across 4 years (2016–2019; Fig. 1). As our models required temporally regular locations, we linearly interpolated the tracks to an interval of one hour.

### Geomagnetic data

**Fig. 2:**
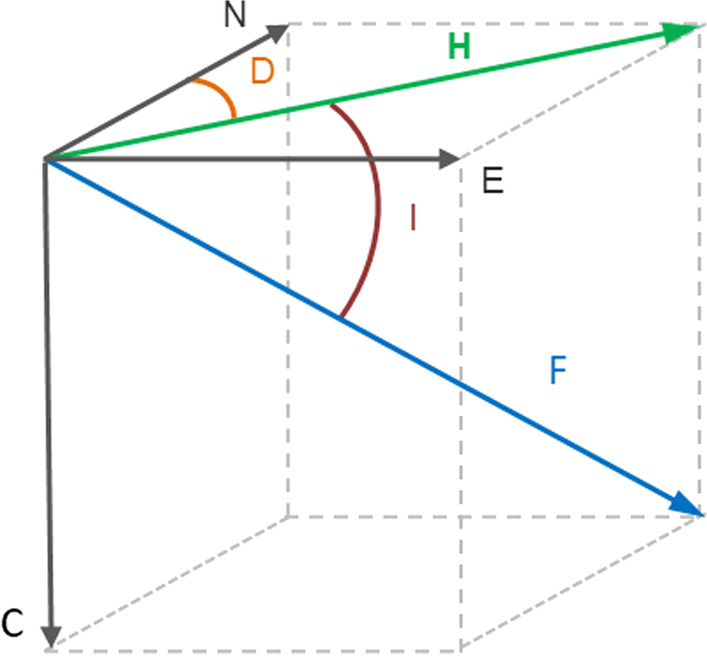
Overview of the Earth’s magnetic-field vectors. The geomagnetic intensity (F) can be represented by a vector in a 3-dimensional plane (geographic north (N), geographic east (E), down (C)). Its component in the N–E plane is the horizontal component (H). The angle between the field intensity and the horizontal plane is the inclination (I) and the angular difference between the geomagnetic and the geographic north is the declination (D)

The Earth’s magnetic field is a bipolar magnet and the magnetic poles are at distance to the geographic poles located at the rotational axis at the Earth’s surface [4]. The magnetic field is a three-dimensional vector, typically measured in the North-East-Centre (or Down) coordinate system (Fig. 2). Magnetic intensity (F) is the length of the field vector and is highest at the poles (60,000–65,000 nT) and decreases towards the magnetic equator (23,000–25,000 nT). The horizontal intensity (H) of the geomagnetic field vector is a strength component based on the north and east components of the geomagnetic field [4]. Magnetic inclination (I) is the angle of magnetic field lines relative to the surface of the Earth [4]. At the magnetic poles, the field lines go straight into the Earth (90°) and are parallel to the Earth’s surface at the magnetic equator (0°). The field is a composite of several components: the core field, generated by the dynamo of the Earth’s core, the lithospheric field, generated by the magnetism of the rocks on Earth’s surface and the field generated by external influences, primarily those by Solar wind [57]. The core and lithospheric fields change slowly, over scales of years to millennia [11]. Solar wind, however, can create disturbances of the field at temporal scales of minutes to hours, in particular during periods of high activity on the Sun, which lead to the so-called geomagnetic storms. Such disturbances may cause rapid changes in geomagnetic field values that the birds can sense and may therefore affect their navigation.

Terrestrial and satellite measurements of the geomagnetic field are used to create global magnetic models, which represent the values of the field at each location on the Earth’s surface at a high temporal frequency. These models are based on real-time Earth’s magnetic field measurements, obtained by the global network (the International Real-time Magnetic Observatory Network, INTERMAGNET) and by a suite of specific geomagnetic satellites (e.g., the Swarm constellation of the European Space Agency). In this paper we used daily modelled geomagnetic data, which are publicly available from the National Centres for Environmental Information (NCEI) of the National Oceanic and Atmospheric Administration (NOAA). These models are based on the 12th Generation International Geomagnetic Reference Field released by the International Association of Geomagnetism and Aeronomy (IAGA) [58] and represent daily changes of the field [58].

**Fig. 3:**
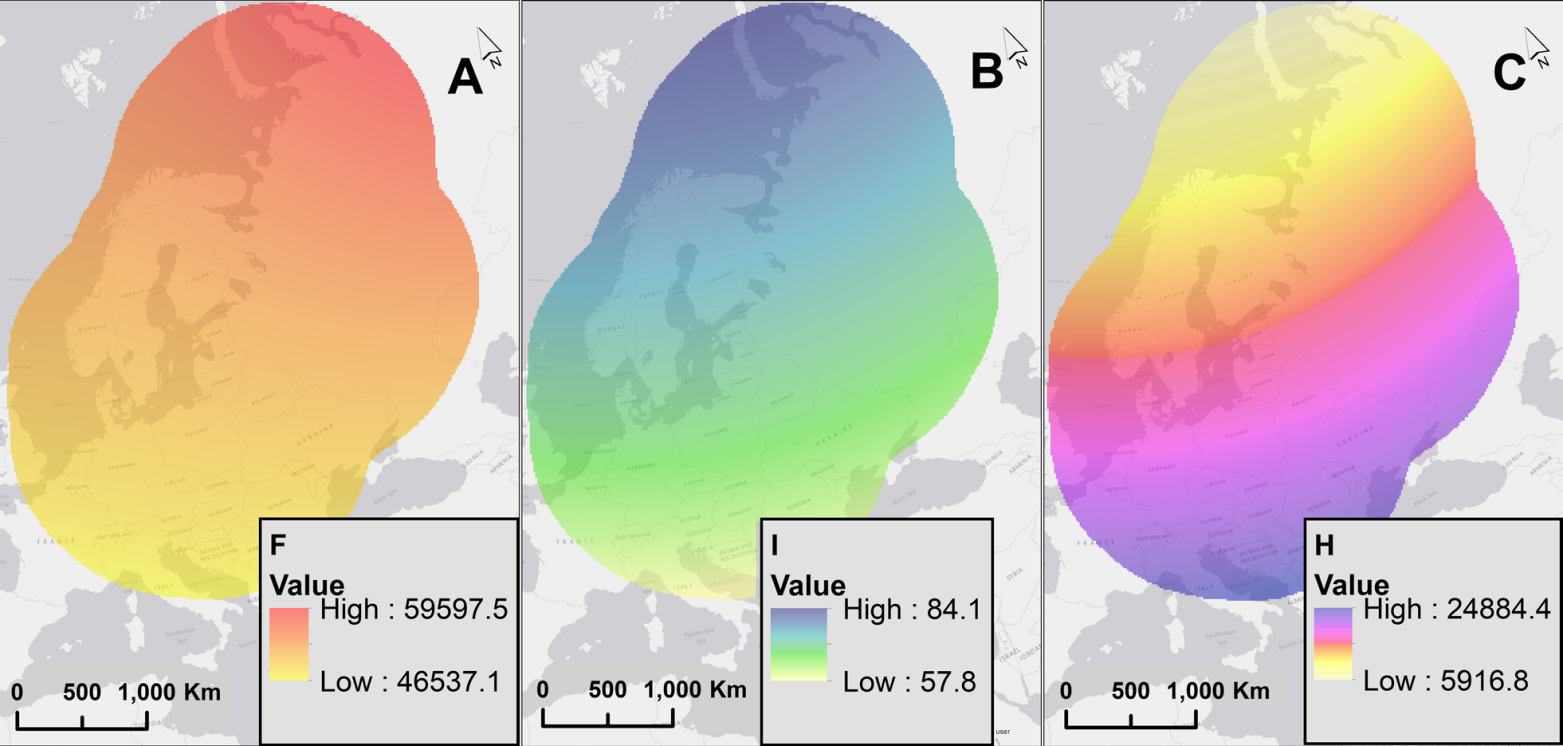
Example maps of the three geomagnetic quantities used in our simulation: (**a**) the intensity F (nT), (**b**) the inclination I (degrees), and (**c**) the horizontal component H (nT), for September 19, 2016

We downloaded modelled data for the area covering our study for every day associated with our GPS tracking data. We retrieved data for magnetic intensity (F, nT), horizontal intensity (H, nT), and magnetic inclination (I, degrees). The IAGA geomagnetic data are obtained at a spatial resolution of 0.1 arc-seconds, which we resampled to a 5 km spatial resolution in the lambert conformal conic projection with parallels at 75- and 50-degrees latitude (and this lambert conformal conic projection was used in all subsequent analyses, Fig. 3).

### Modelling

**Fig. 4:**
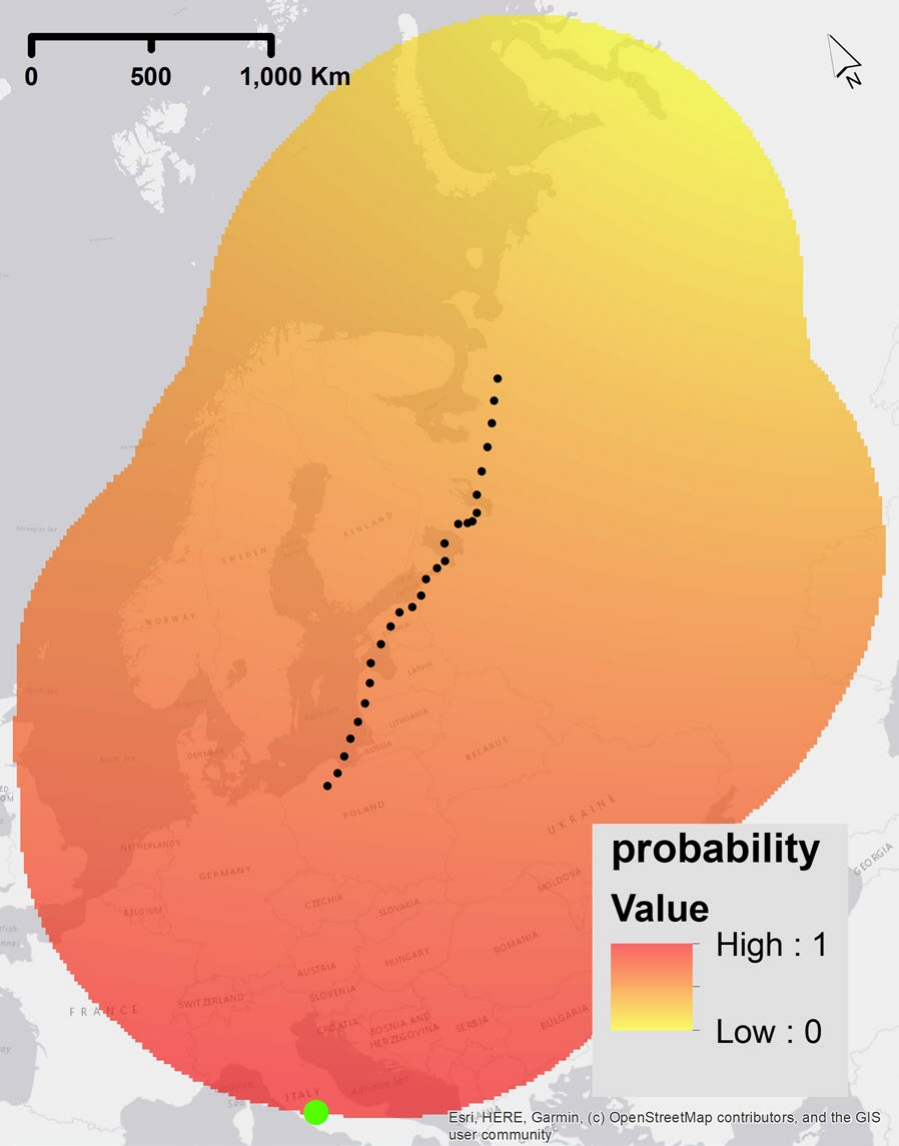
Example of a probability raster for one step of the simulation experiment for the geomagnetic navigational strategy ‘geomagnetic taxis’. The highest probability values are shown red (indicating higher probability of movement steps in that location) and lower probability values are yellow (indicating a lower probability of movement step in that location). These rasters were calculated before each step of the simulated trajectory and are dependent on the current position of the simulated bird and the current condition of the geomagnetic field -the green dot is the location of the minimum value of the raster. The true migratory trajectory of animal 1, interpolated to 1 h frequency, is represented by black dots. We used the geomagnetic field value intensity in this example plot

Birds may rely entirely on geomagnetic navigation or alternatively use a multifactorial navigational mechanism (Fig. 4). To incorporate this in our models we simulated different navigational strategies using correlated random walks (CRW, Additional file 1: Table S1) and correlated random bridges (CRB, Additional 1: Table S1). The CRW models enabled us to simulate models that were a representation of navigation entirely based on geomagnetic navigation. In the CRW models, there was no fixed endpoint of the modelled trajectories and the only bias towards reaching the target location (the end point of each GPS trajectory) was based on geomagnetic navigation. The CRB models are a special case of the CRW [59] where both the start and the end point are fixed in space [50] and in our case were identical to the origin and destination points from the migration defined by the GPS data. This type of model implies additional knowledge during navigation, which is defined by the pull to the target. However, there was still a random component in the simulated trajectories of the CRB models and including geomagnetic navigation could potentially decrease the variation in these simulated trajectories and help us understand the extent and type of geomagnetic information used during these particular journeys.

Correlated random walk (CRW) models simulate movement using pre-defined step length and turning angle distributions (Additional 1: Table S1). In a CRW, the direction in a current step is correlated with the direction in the previous step [53]. In each successive step, the step length and turning angle were drawn from the corresponding distribution. We derived distributions for step lengths and turning angles from the GPS tracking data and pooled step lengths and the turning angles for all individuals. These distributions were used to derive a two-dimensional probability distribution and a unidimensional distribution of the differences of step lengths and turning angles with a lag of 1 to maintain autocorrelation in each of the terms. The additional procedure to look at the autocorrelation of the steps ensured that the trajectories maintained a closer geometrical similarity to the original GPS tracking data. A correlated random walk was calculated with the step lengths and the turning angles and their autocorrelation to generate sufficient input data for the models. Additionally, we set each initial heading to a navigation-specific optimum heading with a turning error of Pi/12 for the CRW models.

A CRB is a correlated random walk were both the start and end point are fixed in space [50]. The pull to the target was derived from the correlated random walk, which was calculated for each step as the distribution of distance to target and the directions to the target [60, 61].

Here, the tracks in our simulated models were generated based on the distributions of step lengths and turning angles observed in the empirical bird migratory tracks. These movement parameters were used to define a probability surface that represents the probabilities of subsequent steps made in the CRW and CRB models. We then combined the probability surface based on the movement parameters (step-length and turning angle) with a probability surface defined by navigational strategies associated with geo-magnetic values (Additional 1: Figure S1). Through this approach it was possible to model realistic steps that captured preference for certain environmental conditions; in this case, preferences associated with different navigational strategies associated with the Earth’s magnetic field.

To estimate a realistic weight between the two probability surfaces, based on the movement parameter and on the environmental (geomagnetic) bias, we re-ran the analysis using other weighting schemes which weigh the geomagnetic navigation strategy differently. These results showed that the weighting influences the simulations in a predicted way with high weightings on the navigation strategy resulting in simulations that are highly deterministic but less related to the geometry of the tracked GPS trajectories of the birds (Additional 1: Table S2, Figure S2). Low weightings resembling true correlated random walks (Additional 1: Table S2). For our chosen models we took the square root of the movement probability raster multiplied by the bias probability raster of the geomagnetic strategies. This enabled us to put a higher weight onto the geomagnetic bias while still maintaining the movement parameters of the tracked birds.

Each navigational strategy involves different calculations applied to the geomagnetic data to create the probability surface that was used to condition the random walk models. Based on literature we modelled five different navigational strategies including a control without geomagnetic bias (*no bias*), simulated as CRW and CRB models with no conditional component. In the following section we describe the other four navigational strategies, followed by an explanation of how we incorporated these into our simulation models. The models are explained in more detail in Additional 1: (S1).

### Geomagnetic taxis

Geomagnetic taxis is the navigational strategy based on a bird flying towards a single global geomagnetic extreme along a gradient [36, 62]. This means that the bird is following along one gradient, but with no defined end point. Equally, this could mean a bird is constantly flying towards a local extreme value in the geomagnetic field (e.g. heading towards the geomagnetic north or south). Given that this is migratory movement, and since we focus on autumn migration (in this case from high to low geomagnetic values), the global minimum of the magnetic field was modelled as having the highest probability of movement (1) and the maximum magnetic value the lowest probability (0, Fig. 4). We used linear scaling to model the probability of selecting a location relative to the maximum and minimum values. For navigation, this means that if the desired magnetic value (e.g., the minimum) was in line with the migratory path, maximising the sensed values of the magnetic field can be used to reach a target destination. We repeated this process with each of the different geomagnetic properties F, I, and H.

### Map-constant heading

**Fig. 5:**
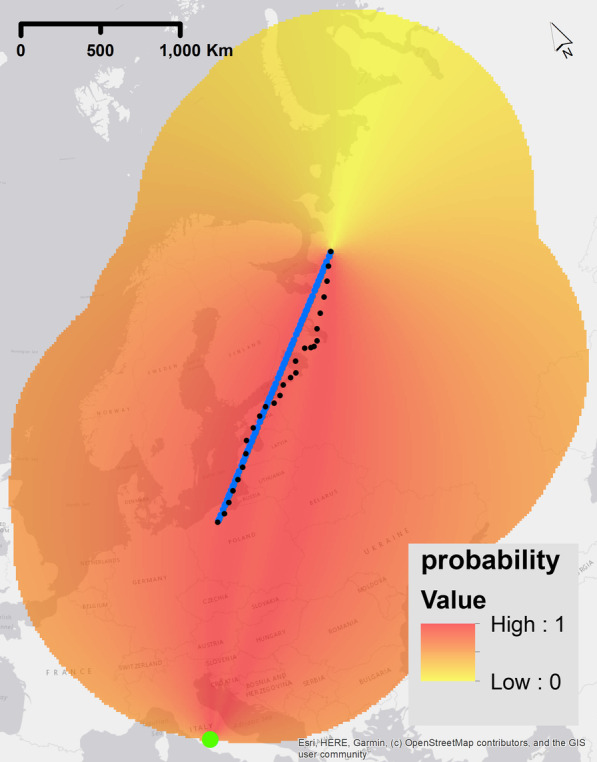
Example of a probability raster for one step of the simulation experiment for the geomagnetic navigational strategy ‘Map-constant heading’. The highest probability values are shown red (indicating higher probability of movement steps in that location) and lower probability values are yellow (indicating a lower probability of movement step in that location). These rasters were calculated before each step of the simulated trajectory and are dependent on the current position of the simulated bird and the current condition of the geomagnetic field -the green dot is the location of the minimum value of the raster. The true migratory trajectory of animal 1, interpolated to 1 h frequency, is represented by black dots. The blue line represents a straight line between the current position of the simulated trajectory at the start of the migration and the final point of the GPS trajectory. We used the geomagnetic field value intensity in this example plot

Similar to flying towards a global geomagnetic extreme, the extreme values might be used as a point of reference for navigation. This navigation could be used to keep a constant heading/constant angle towards a geomagnetic cue [4, 24–26, 63]. Constant heading is another form of compass navigation, where instead of following the gradient, an extreme value is used to maintain a constant heading. However, if a bird uses constant heading based on memory of a previous migration, then constant heading could be classified as compass navigation alone. However, since the earth magnetic field values are constantly changing this is not likely to be the case. Therefore, to navigate with constant heading compass navigation, map navigation is required for positioning in relation to the target location and daily calibration to geomagnetic changes. In our study, the bird calibrates the constant heading based on the angle between target position and extreme values of the geomagnetic field, therefore we define constant heading, in our case, as map navigation. We calculated the probability raster where the maximum value was the mean angle of the bird’s straight-flight trajectory with respect to the extreme geomagnetic value. First, the angle between the extreme geomagnetic value, the current position of the simulated bird at the start of each day, and any point on raster were calculated. The angle was calculated for one daily position only because biologically most evidence suggests towards one calibration a day in birds [7, 64]. For the probability raster the mean angle of all points on the line between the start/current position and the end point of the trajectory had the highest probability. Angles higher or lower were calculated according to 180 degrees = 100%. The model was run twice for each geomagnetic value (F, I, H), with the global geomagnetic minimum and with the global geomagnetic maximum. Figure 5 shows an example probability surface for this strategy.

### Map-bi-gradient taxis navigation

**Fig. 6:**
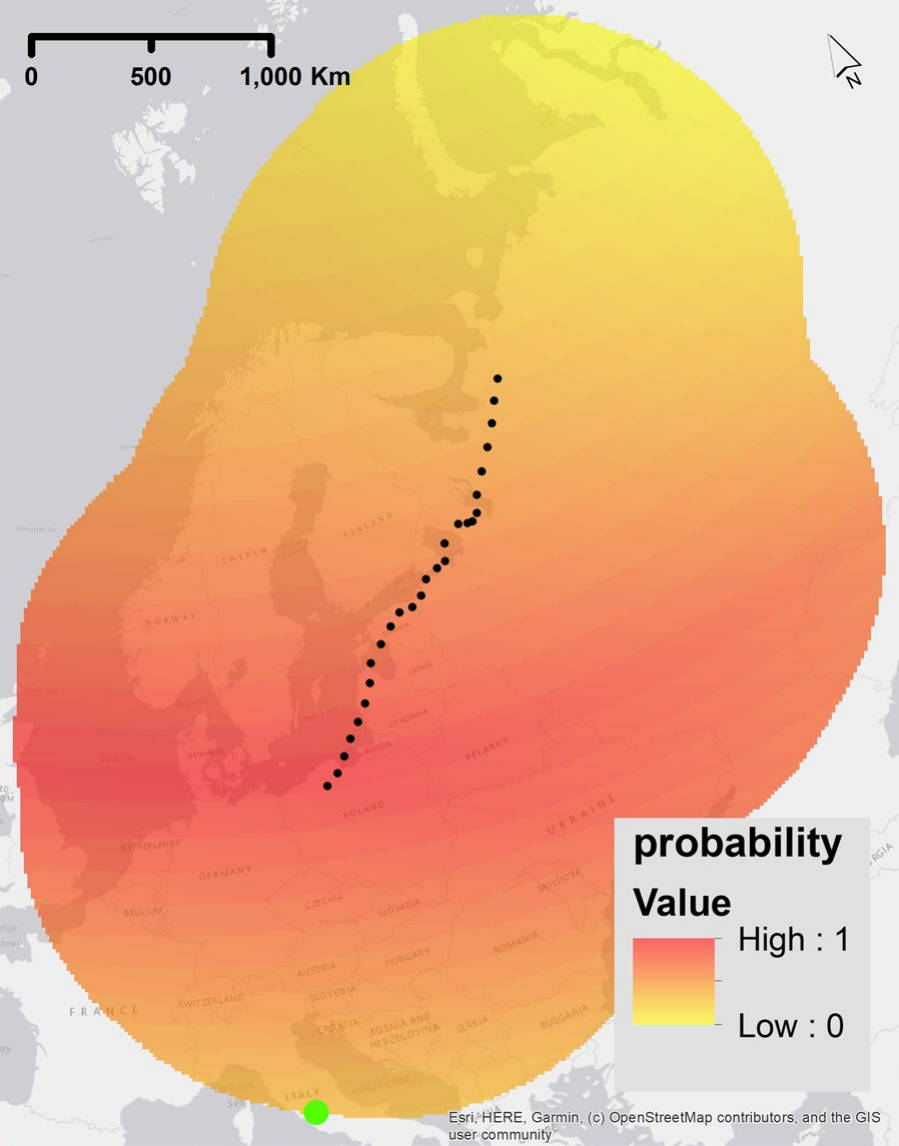
Example of a probability raster for one step of the simulation experiment for the geomagnetic navigational strategy ‘Map-bi-gradient taxis navigation’. The highest probability values are shown red (indicating higher probability of movement steps in that location) and lower probability values are yellow (indicating a lower probability of movement step in that location). These rasters were calculated before each step of the simulated trajectory and are dependent on the current position of the simulated bird and the current condition of the geomagnetic field -the green dot is the location of the minimum value of the raster. The true migratory trajectory of animal 1, interpolated to 1 h frequency, is represented by black dots. We used the geomagnetic field value intensity in this example plot

Birds could navigate based on a two-dimensional geomagnetic map. Map navigation or map-based orientation is based on the knowledge of values that can be sensed for navigation at a target location and a navigation towards these values [30–34]. In theory, this form of map navigation is geomagnetic taxis navigation but along two different gradients, however an additional difference is the clear positioning towards an end value of the target location. Therefore, two different values are needed for orientation that were derived separately from the geomagnetic values. For both, the probability of 1 was the geomagnetic value at the end point of the trajectory. The full range of probabilities was calculated with the range of the geomagnetic values in each raster with linear scaling, similar to the process for geomagnetic taxis. The probability rasters were calculated separately for each of the two geomagnetic values used and multiplied (Fig. 6). Different combinations of magnetic values were modelled as per the following: F–I, F–H, I–H.

### Combination map bi-gradient taxis - map constant heading

**Fig. 7:**
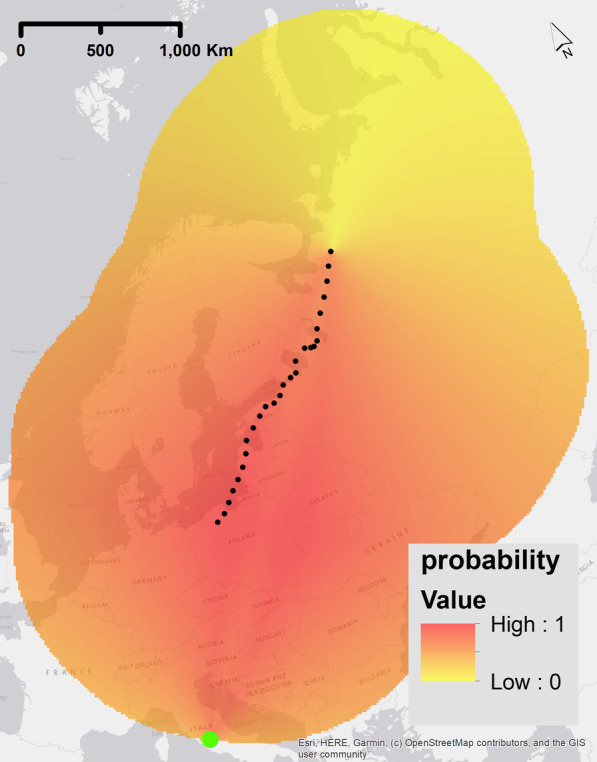
Example of a probability raster for one step of the simulation experiment for the geomagnetic navigational strategy ‘combination bi-gradient taxis - constant heading’. The highest probability values are shown red (indicating higher probability of movement steps in that location) and lower probability values are yellow (indicating a lower probability of movement step in that location). These rasters were calculated before each step of the simulated trajectory and are dependent on the current position of the simulated bird and the current condition of the geomagnetic field -the green dot is the location of the minimum value of the raster. The true migratory trajectory of animal 1, interpolated to 1 h frequency, is represented by black dots. We used the geomagnetic field value intensity in this example plot

Combinations of map navigation with other navigational strategies have also been suggested [24, 33–38]. An example of the combination of different navigational strategies could be a combination of map navigation and compass constant-heading strategy. We calculated two different probability rasters, one based on constant heading navigation and one based on map navigation (see above). Both final rasters were multiplied (Fig. 7) and different combinations based on the geomagnetic values were modelled: F–I, I–F, F–H, H–F, I–H, H–I.

Table 1 provides an overview of the different navigational strategies and associated geomagnetic values. CRWs and CRBs were calculated for every navigational strategy [5] and repeated for all 3 magnetic values (intensity F, inclination I and horizontal component H) independently or in combination which led to 19 different models for simulation. Each simulation model was repeated to generate 100 simulations per model and the whole process was repeated for all 14 autumn migratory flights, resulting in 1400 simulations per model. Some strategies had multiples of 1400 simulations, depending on how many geomagnetic values or combinations were used.

**Table. 1:** List of all strategy combinations in association with the geomagnetic values. These 19 combinations were used for the CRW and CRB models

Strategy	Geomagnetic values used in models						Total number of simulations
1. No bias	None						1400
2. Geomagnetic taxis	F	H	I				4200
3. Constant heading	MaxF	MaxH	MaxI	MinF	MinH	MinI	8400
4. Bi-gradient taxis	FH	FI	IH				4200
5. Combination bi-gradient taxis-constant heading	FH	FI	HF	HI	IF	IH	8400

### Statistical analysis

For each migratory flight and each type of navigation we compared the simulated trajectories derived to the real trajectories using three measures of trajectory similarity (Ranacher and Tzavella, 2014): the mean distance [65], the dynamic time warping [66], and the dynamic interaction index [67]. We calculated the mean distance of every point of the simulated trajectory in relation to the corresponding location in the matching empirical trajectory. Lower values of the mean distance are reflective of more similar movement. Dynamic time wrapping (dtw) accounts for the variability of distances (e.g., the mean is sensitive to outliers). This method dynamically adapts the simulated trajectory to the appearance of the GPS- trajectory, by shifting and stretching and through this correcting for incorrect comparisons [66]. Equally to mean distance, lower values of the dtw are reflective of more similar movement. The final measure we computed was the dynamic interaction (DI) index, which is a measure of similarity in the direction and displacement components of movement steps [67]. DI values near 0 suggest no similarity, whereas values close to 1 indicate high similarity of movement.

To test if any of the simulated navigational models differed from the one without geomagnetic input, we calculated linear mixed effect models. Values of the 3 different similarity measurements were modelled separately against the different simulated strategies. To account for autocorrelation, introduced by repeated measures, we added a random intercept effect term for animal identity. These 3 linear mixed effect models were calculated for CRW and CRB separately.

To evaluate and count the number of the best performing simulations in our models we needed to account for the fact that different strategies were associated with a different number of model simulations. Therefore, we used the linear mixed effects models to strategically choose the specific geomagnetic cue (i.e., I, H, or F) that best represented each of the five strategies in Table 1. For example, for geomagnetic taxis we selected the model using geomagnetic intensity, based on comparison of the estimate output of each model (Additional file 1: 3). Thus, for each of the five strategies we retained an equal number of 1400 simulations. These 1400 simulations (× 5 strategies) were then pooled to create an overall sample of 7000 simulations. To evaluate our simulations and acquire the best performing strategy and geomagnetic characteristic, we selected the top 10% of the 7000 simulations (following Oudman et al., 2020) and counted the occurrences of the different geomagnetic strategies for each similarity measure. The proportion of the 10% of the simulations containing each of the geomagnetic strategies is then used as evidence of which model more closely resembled the true movement data.

All computation was done in R 3.6.3 using the following packages: move [69] and adehabitatLT [70] to analyse trajectories and lme4 [71] to fit the statistical models.

## Results

### Navigation strategies: simulations based on taxis navigation are the most similar to GPS trajectories

**Fig. 8:**
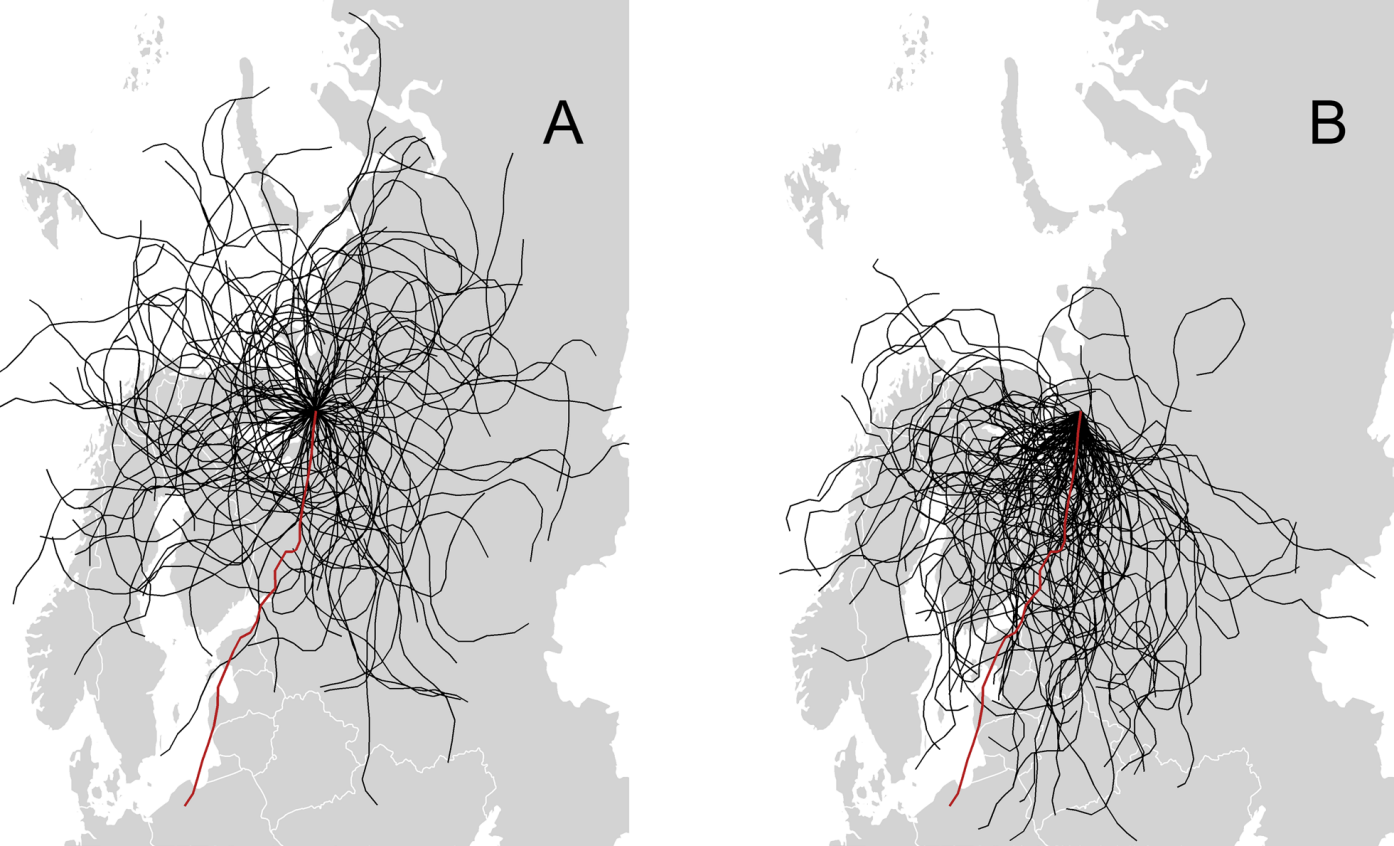
An example CRW simulation for animal 1. **a** The simulation without geomagnetic bias and **b** the simulation with geomagnetic taxis for intensity F

For every bird we simulated 5 different navigational strategies with different combinations of the geomagnetic values with CRW (an example is presented in Fig. 8, while the full output is in Additional 1: 2, Table S3, Figures S3–S16, S3 Model 1-3). In the CRW models, the strategy based on taxis navigation was the one most often represented in the sample of the top 10% of the simulations. The count of the taxis navigation in the best models was 31–32% for all three similarity measurements (Table 2). About 20% of counts, which is the amount expected by equal distribution of the 5 strategies, was found for the navigational strategies based on constant heading and the combination of bi-gradient taxis and constant heading. The least similar of the simulated trajectories used no additional bias and bi-gradient taxis navigation and accounts for 9–17% of the counts.

**Table. 2:** Counts and proportions of navigational strategies in the 700 best simulation runs for each of the three trajectory similarity measures (mean, dtw, DI)

Strategy	Mean		dtw		Similarity	
	Count (#)	Proportion (%)	Count (#)	Proportion (%)	Count (#)	Proportion (%)
CRW						
No additional bias	65	09	74	11	100	14
Geomagnetic taxis	226	32	224	32	220	31
Constant heading	155	22	149	21	131	19
Bi-gradient taxis	110	16	118	17	103	15
Combination Bi-gradient taxis -Constant heading	144	21	135	19	146	21
CRB						
No additional bias	127	18	123	18	139	20
Geomagnetic taxis	153	22	113	16	140	20
Constant heading	134	19	150	21	150	21
Bi-gradient taxis	145	21	152	22	136	19
Combination Bi-gradient taxis -Constant heading	141	20	162	23	135	19

### Geomagnetic parameters: navigation based on geomagnetic intensity and inclination are most similar to GPS trajectories

Navigational simulations were based on three different geomagnetic values. The geomagnetic intensity (F) and inclination (I) were represented in more than 50% of the best models in the two geometric output statistics mean and dtw in CRW models (Table 3). In the similarity measurement F is represented in 86% of the best models. This shows that simulations based on F were most similar to the original trajectory. The horizontal geomagnetic component (H) was used in 21% of the best models in the similarity measurement, however H was only included in combination with F (Table 3).

**Table. 3:** Counts and probabilities proportions of geomagnetic cues in the 700 best simulation runs for each of the three trajectory similarity measures (mean, dtw, DI)

Strategy	Mean		dtw		Similarity	
	Count (#)	Proportion (%)	Count (#)	Proportion (%)	Count (#)	Proportion (%)
CRW						
No additional bias	168	24	168	24	100	14
F	93	13	160	23	351	50
FI	290	41	235	34	103	15
FH					146	21
I	149	21	137	20		
CRB						
No additional bias	130	19	143	20	139	20
F	282	40	144	21		
HF	145	21	131	19	135	19
I			140	20	290	41
IH	143	20	142	20	136	19

### CRB simulations

**Fig. 9:**
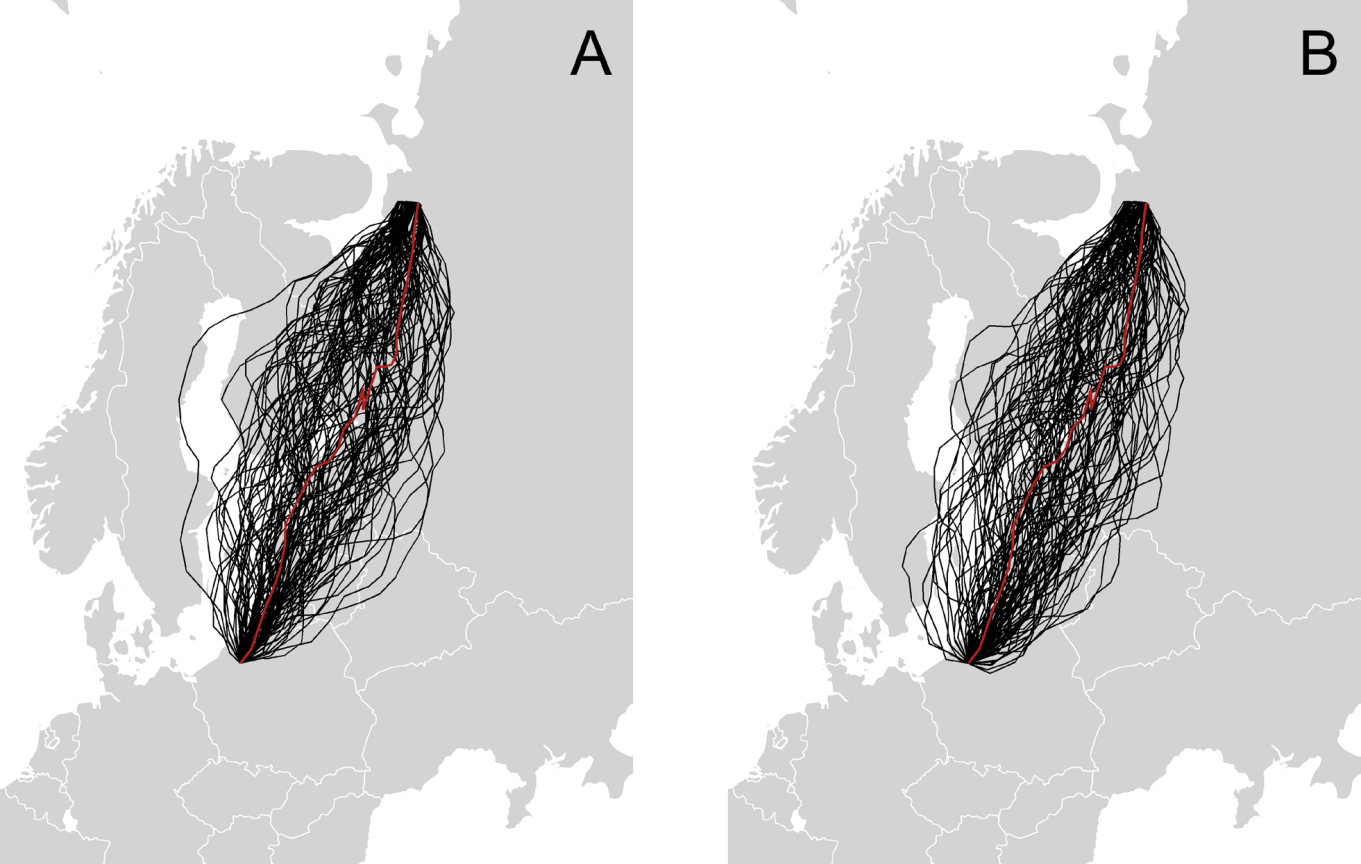
Example CRB simulation of animal 1. **a** The results of the strategy with no magnetic bias and **b** with geomagnetic taxis and associated geomagnetic value F

For the CRB models the counts of the 5 different navigational strategies of the top 10% sample of the best model did not change (an example in Fig. 9 and full output in Additional 1: 2, Table S3, Figures S17–S30, S3 Model 4-6). In all three similarity measurements all 5 simulation strategies were represented equally at around 20% (Table 2). Similarly, there was no clear pattern when comparing the use of the geomagnetic values in the CRB models (Table 3). There was a higher count of simulations using navigational strategies with F and H, for the mean distance and dtw measurements and contrary to this, a higher count of I navigational simulations was found in the similarity measurements (Table 3).

## Discussion

Our results indicate that simulations based on underlying gradients that minimise, in the case of autumn migration, geomagnetic values (taxis), were more likely to produce migratory trajectories that were most similar to the observed geese migratory trajectories. This finding could indicate that, under the modelled geomagnetic conditions, this navigational strategy would be the most likely when used independently from other cues. However, it was not probable that the simulated trajectories end up in the target locations even when strongly biasing step selection based on the probability raster for geomagnetic taxis. Based on our simulations, the most likely geomagnetic navigational strategy for the birds would be to use geomagnetic taxis as a general directional tool but they clearly would need additional navigational cues, like landmarks, sun and stars, odour, or sound for more precise navigation decisions when using geomagnetic navigation [4, 5, 72–74]. Thus, our data-driven approach supports previous theories of multifactorial navigation [4, 5] especially for long-distance migration.

The geomagnetic properties with the best modelled output in the CRW models were based on the field intensity (F) and inclination (I). Thus, if birds were following a geomagnetic gradient, like in geomagnetic taxis, then following geomagnetic cues which are in-line with the target destination would be most accurate. One difference between intensity F and inclination I is the spatial dynamics of changes in these values and the birds’ ability to sense these changes. Geomagnetic intensity changes globally on average 10nT/km whereas inclination changes at a rate of 0.009°/km. The simulated birds could be influenced by the value which changes at a finer spatial resolution, i.e. F. In comparison between the spatial scale of variation of the geomagnetic field and the level of geomagnetic sensitivity of birds (F ~ 20nT [17] I close to 1° [23]), a bird would need to fly at least 2 km to detect a change in intensity F and 112 km to detect a change in inclination I, which indicates that navigation based on F is possible on finer spatial scales. Despite a lower spatial scale of change of I, it still might be a useful tool for broad navigation on long-distance migratory flights and could function as a backup if other navigational cues are not available. Additionally, the broader spatial resolution of inclination might make navigation depending on it less affected by local and temporal changes of the geomagnetic field and therefore a more reliable geomagnetic navigation value. These observations affect the plausibility that birds are using these different cues and indicate that the use of different geomagnetic cues (F or I) might vary depending on local conditions.

In our simulations we found that modelling geomagnetic navigation as a correlated random bridge (CRB) is mathematically possible but does not lead to any difference in outputs for different navigational strategies. A main feature of these models is the strong pull to the target destination [75] without any underlying navigation or orientation mechanism underlying or justifying it. This is a very strong bias which overrides the comparatively weak signal of the geomagnetic navigation effect. In theory, a stronger effect of geomagnetic navigation could have manifested in the simulated trajectories being more similar to the original bird trajectory even in CRB models. The absence of such effects in the results indicates that the effect of the geomagnetic navigation of our simulations is relatively weak compared to the pull to the target as modelled in a CRB. For the bird navigation example studied here, this could mean that geomagnetic navigation might play a minor role in the multifactorial navigational mechanism of these geese. If the pull towards the target is eliminated, as in the CRW models, the independent effect of the geomagnetic field values on the navigation during migration becomes more apparent, but not to such an extent that it would permit to successfully arrive at the target without additional non-geomagnetic cues.

All geomagnetic values were based on modelled data, which represent a daily variation of the geomagnetic field albeit only as a rough representation of reality. The temporal dynamics of the geomagnetic field are much more pronounced, as fluctuations at the biologically relevant scale occur at much higher temporal resolution [76]. The geomagnetic field varies over short timescales through the effect of solar winds. Eruptions on the Sun affect the Earth’s magnetic field and during geomagnetic storms induced by the strong solar wind, disturbances may range up to 1000 nT in intensity in polar regions and 250 nT in mid latitudes [57], well in excess of what birds can sense. These changes can occur over periods of seconds to hours but are not visible in the modelled geomagnetic data we used here [77]. Geomagnetic storms occur most frequently around both equinoxes [78], which corresponds with spring and autumn migrations of many bird species. Our modelling could therefore be further improved with the use of directly observed geomagnetic data that are sampled at higher temporal frequencies. Including high-resolution geomagnetic dynamics would improve simulations of migratory flights and lead to better insights into the real conditions experienced by migrating birds through comparisons with the increasing volume of available migratory tracks of birds in the wild.

As we are introducing a new methodology to study geomagnetic navigation from contemporaneous magnetic data with biased random walks, we encountered theoretical limitations. One was related to the choice of weighting of the rasters representing the properties of the animal movement (persistence) and the local geomagnetic conditions (bias). In statistics, there is no optimal method of how to combine these two properties. Instead, for each case study, it is necessary to make simplifications to model equations or assumptions as to how organisms respond to variability in their environment [53]. In our case, we derived the weights for persistence and bias rasters based on tests of several weighting schemes. We chose the one that was generating the most stable results, but we acknowledge that this simplification could have been done in a different way. Choosing weights for biased random walks is a complex operation and could be considered as a methodological problem on its own.

A related methodological issue is why simulated trajectories often resemble unbiased Brownian motion rather than being grouped into a narrow corridor around the actual trajectory. There are several possibilities as to why this may be the case. One could be that movement parameters persist for longer than we accounted for. Our model considers an auto difference structure of one step, i.e. it maintains the empirical change in parameters between consecutive steps, but this does not extend to the following steps. To represent a longer persistence, the model could be adjusted to include a longer sequence of steps, but that would increase computational complexity as well as complicate interpretation. Second, the large geographic variability in simulated trajectories could be an indication that geomagnetic navigation is not prioritised by birds as the primary navigation mode. This is supported by the fact that birds use multifactorial navigation mechanisms [4, 5], which we however do not account for in our models. Combining several navigational strategies into a spatio-temporal mathematical model is a very complex undertaking. A major hurdle is calculating similar raster surfaces that account for other navigation strategies. For example, another proposed strategy for migratory bird navigation across featureless expanses is olfactory navigation [79]. It is at present not possible to study olfactory navigation in a modelling approach such as the one presented here, since data on atmospheric concentration of the volatile organic compounds (which are hypothesised to guide the birds) are not available at geographic extents and sufficient spatial and temporal resolutions that would be suitable for analysing long-distance migratory flights. Our paper therefore focuses on single cue navigation as we are limited to data that does exist (on Earth’s magnetic field) but this could be expanded in the future, as new environmental data become available. Finally, in this experiment we used modelled geomagnetic data, which, as discussed above, may not represent the actual magnetic environment during disturbed geomagnetic conditions and may therefore impart less pull on the simulations. We plan to address this problem in our future work, where we will use accurate contemporaneous geomagnetic measurements, derived from satellite sources.

Geomagnetic bird navigation has been studied using many different approaches in the past [10, 17–23, 39–44], and these studies do not always agree on how geomagnetic navigation might occur, especially during migration. Current studies are limited in that they use methods that are difficult to compare, and typically only study some navigation strategies or geomagnetic values (Intensity or Inclination). Thus, the current body of literature is limited in terms of the overall conclusions which can be drawn about how geomagnetic navigation might occur in migratory birds. Additionally, geomagnetic navigation is likely species- and/or individual specific, dependent on the location on the Earth and variations in the geomagnetic field. Our simulation experiment can be applied to different species, incorporates geomagnetic data at relatively fine spatial and temporal resolution, and can integrate various navigation strategies. This allows for direct comparison between species, strategies, and at different locations globally.

## Conclusions

In this paper, we present a data-driven simulation experiment to evaluate which geomagnetic navigational strategies are mostly used by geese during fall migration from Russia into Europe. Our approach can now be extended to study navigational strategies of migratory birds in general. In our simulations, navigational strategies based on taxis were the most likely to occur and our results corroborate that migratory navigation is likely a multifactorial process. We showed that the spatial and temporal scales of change in the geomagnetic values as well as the physiological abilities of the birds to sense these values are important factors during navigation. Simulation experiments testing the effect and optimum resolution of geomagnetic input variables are needed to gain a better understanding of the geomagnetic navigational strategies used by birds. Additionally, models could be improved by using more temporally dynamic geomagnetic data, to capture the high temporal variability of the changes of the field, which may affect bird navigation.

## Supplementary Information

**Additional file 1**. This file is containing further explanations of the methods and a methodological workflow (S1), visual results for all analysis (S2) and all statistical outputs (S3).

## Data Availability

The datasets analysed during the current study are available on Movebank.
