# Supplementary material for: Simulation experiment to test strategies of geomagnetic navigation during long-distance bird migration
Source: Mov Ecol. 2021 Sep 15;9:46. doi: 10.1186/s40462-021-00283-5 (PMC8442449; doi:10.1186/s40462-021-00283-5)
Supplement: Supplementary file 1 — Additional file 1. This file is containing further explanations of the methods and a methodological workflow (S1), visual results for all analysis (S2) and all statistical outputs (S3). [file 40462_2021_283_MOESM1_ESM.docx]

**Supplementary Material**

**S1 - Methods**

Modelling

*Table S1. Properties of different random walk models; those marked in grey were models we used in simulations to capture different navigation strategies in migratory birds.*

| Method | Correlation in turning angle | Defined end point | Environmental gradient |
| --- | --- | --- | --- |
| Random walk |  |  |  |
| Correlated random walk (CRW) | X |  |  |
| Conditional correlated random walk | X |  | X |
| Random bridge (also called biased random bridge) |  | X |  |
| Correlated random bridge (CRB) | X | X |  |
| Conditional correlated random bridge | X | X | X |

**Inclusion of environmental raster (conditional random walk)**

In general, environmental information can be included into random walks in each step by generating an underlying probability surface which reflects underlying environmental preferences (1). This two-dimensional probability distribution associated with underlying environmental preferences is then multiplied by the two-dimensional step length and turning angle distribution of the random walk to generate what is termed a conditional random walk (2).


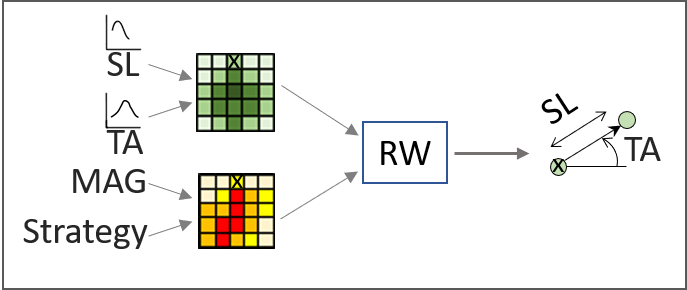
*Figure S1 Methodological workflow: Two different probability raster are combined in the random walk model (RW), the probability surface based on movement parameters (step-length, SL; and turning angle, TA) with the probability surface defined by navigational strategies associated with geomagnetic values (MAG). The output is a realistic simulation of a step with a preference for a certain environmental condition (navigational strategies associated with the Earth’s magnetic field). The x refers to the current position.*

**Determination of weight between probability rasters in the random walks**

We calculated three different weight scenarios for four randomly selected animals (Table S2). We used the weights as follows: 1.) we used the square root from the movement raster (sqrt(move)); additionally, we included an additional bias to the geomagnetic rasters/bias with 2.) the power of 2 (MAG^2) and 3.) with the power of 5 (MAG^5). Even though there is some variation in the model output, it underlines that if the weight towards the geomagnetic strategy rasters is increased the average values from all strategies for that animal are improving (Table S2). However, this also leads to e.g. overshooting of the simulated trajectory and therefore relates less to the original trajectory of the bird (Figure S2). For our methods we decided to include a minimal bias taking the square root of the movement probability raster when multiplying with the bias probability raster. This enables us to put maximum weight onto the bias and still maintaining the movement parameters of the tracked birds.

*Table S2 The average similarity measurements (mean, dtw, sim) were calculated for different weights between the movement probability raster(move) and the geomagnetic probability bias (MAG). We randomly selected four animals (id=1,2,5,9). The yellow fields indicate the “best” model outputs e.g. the lowest values for mean and dtw and the highest values for sim.*

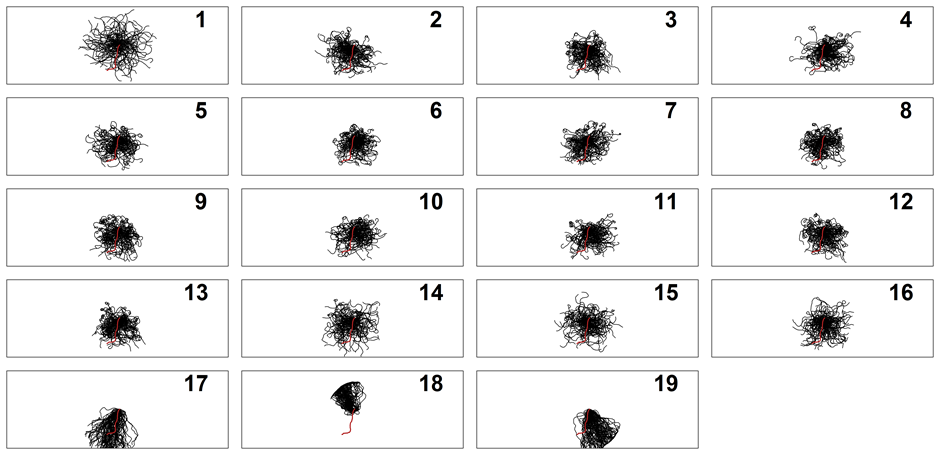
*Figure S2. An example output for the CRW of Animal 8 where the geomagnetic navigation strategies are weight by the power of 5. The sub figures represent the output of the CRW models from 5 navigation strategies with different combinations of geomagnetic inputs (see Table S2 for reference) for animal 1. The labelling is as per the following: No bias (1), Constant heading (2 Max F, 3 Max H, 4 Max I, 5 Min F, 6 Min H, 7 Min I), Combination Bi-gradient taxis-Constant heading (8 FH, 9 FI, 10 HF, 11 HI, 12 IF, 13 IH), Bi-gradient taxis (14 FH, 15 FI, 16 IH), Geomagnetic taxis (17 F, 18 H, 19 I). The simulation based on the taxis strategies (17-19) are overshooting the target and many of the other simulated trajectories are going in circles.*

References

1. Benhamou S. Detecting an orientation component in animal paths when the preferred direction is individual-dependent. Ecology. 2006;87(2):518–28.

2. Turchin P. Quantitative analysis of movement: measuring and modeling population redis- tribution in animals and plants. Sunderland: MA: Sinauer Associates; 1998.

**S2 - Results**

For every bird we simulated 5 different navigation strategies with different combinations of the geomagnetic values (Table S3). This led to 19 different simulations for every bird with CRW models (Figures S3-S16) and additionally for every bird in the CRB models (Figures S17-S30).

Table S3 A list of the simulated models for every bird which explains the numbers in the sub figures below.

1. No bias

Constant heading

1. Max F
2. Max H
3. Max I
4. Min F
5. Min H
6. Min I

Combination Bi-gradient taxis-Constant heading

1. FH
2. FI
3. HF
4. HI
5. IF
6. IH

Bi-gradient taxis

1. FH
2. FI
3. IH

Geomagnetic taxis

1. F
2. H
3. I

**CRW**

Animal 1
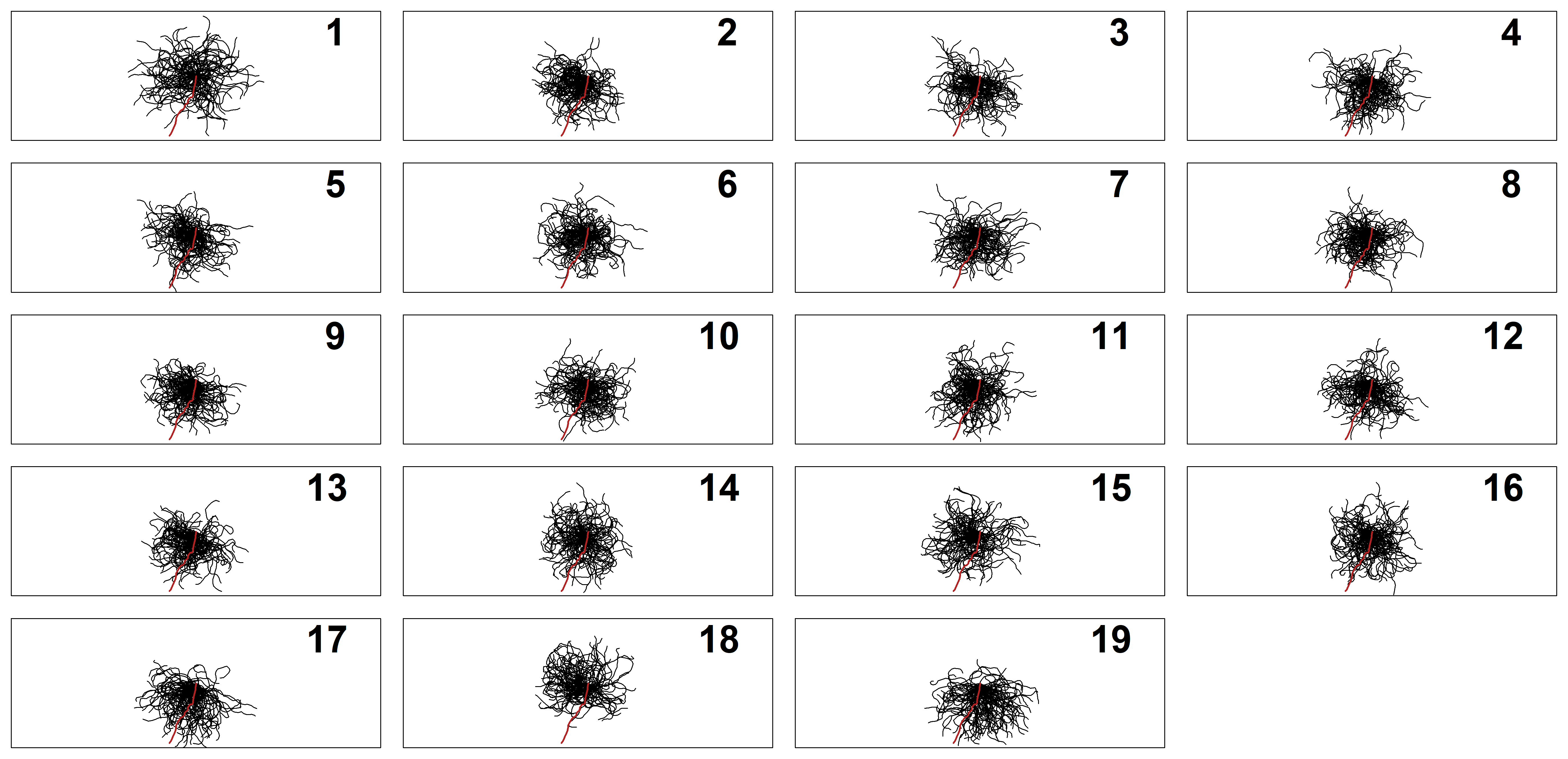


*Figure S3 The sub figures represent the output of the CRW models from 5 navigation strategies with different combinations of geomagnetic inputs (see Table S2 for reference) for animal 1. The labelling is as per the following: No bias (1), Constant heading (2 Max F, 3 Max H, 4 Max I, 5 Min F, 6 Min H, 7 Min I), Combination Bi-gradient taxis-Constant heading (8 FH, 9 FI, 10 HF, 11 HI, 12 IF, 13 IH), Bi-gradient taxis (14 FH, 15 FI, 16 IH), Geomagnetic taxis (17 F, 18 H, 19 I).*

Animal 2
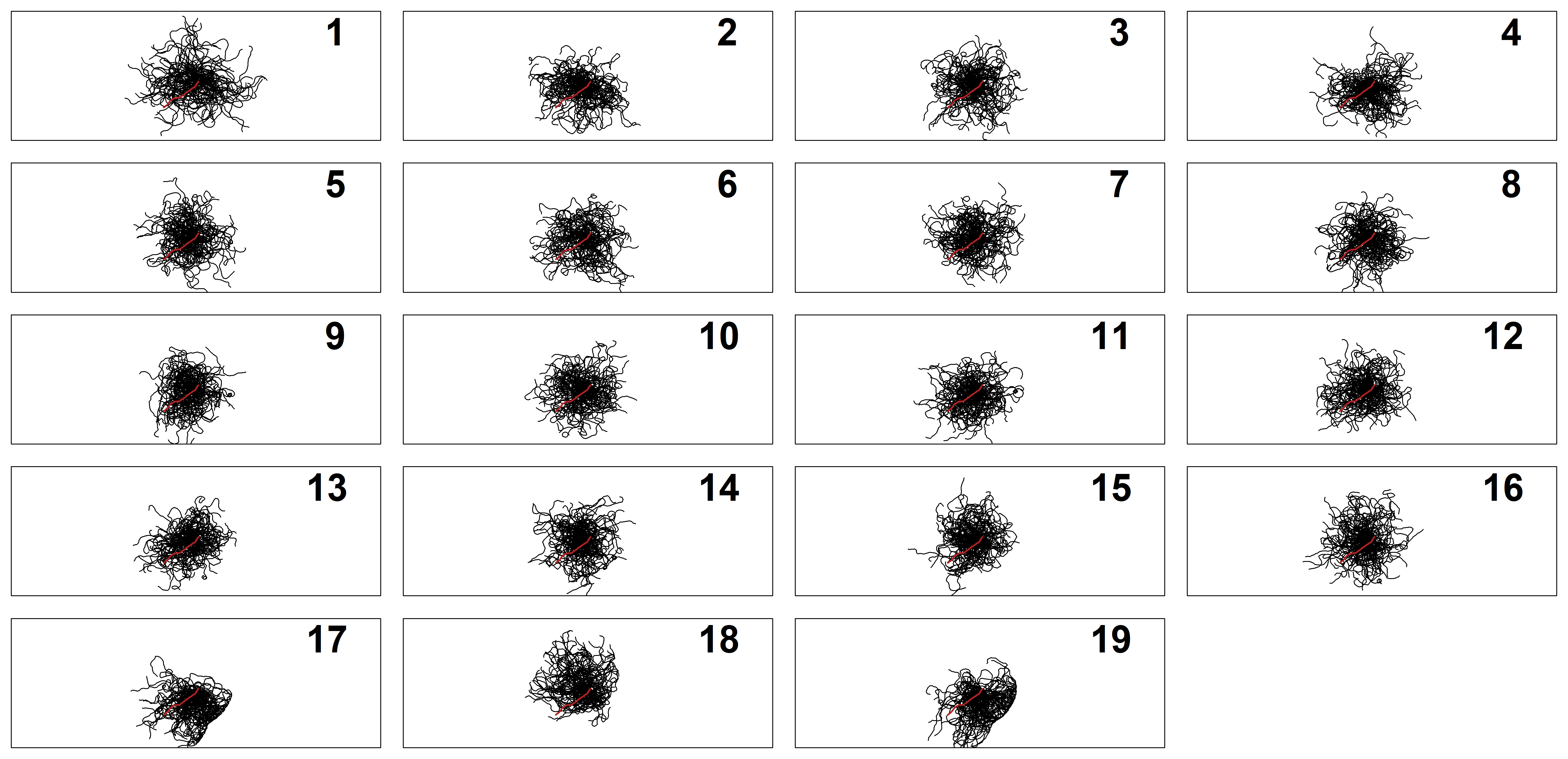


*Figure S4* *The sub figures represent the output of the CRW models from 5 navigation strategies with different combinations of geomagnetic inputs (see Table S2 for reference) for animal 2. The labelling is as per the following: No bias (1), Constant heading (2 Max F, 3 Max H, 4 Max I, 5 Min F, 6 Min H, 7 Min I), Combination Bi-gradient taxis-Constant heading (8 FH, 9 FI, 10 HF, 11 HI, 12 IF, 13 IH), Bi-gradient taxis (14 FH, 15 FI, 16 IH), Geomagnetic taxis (17 F, 18 H, 19 I).*

Animal 3
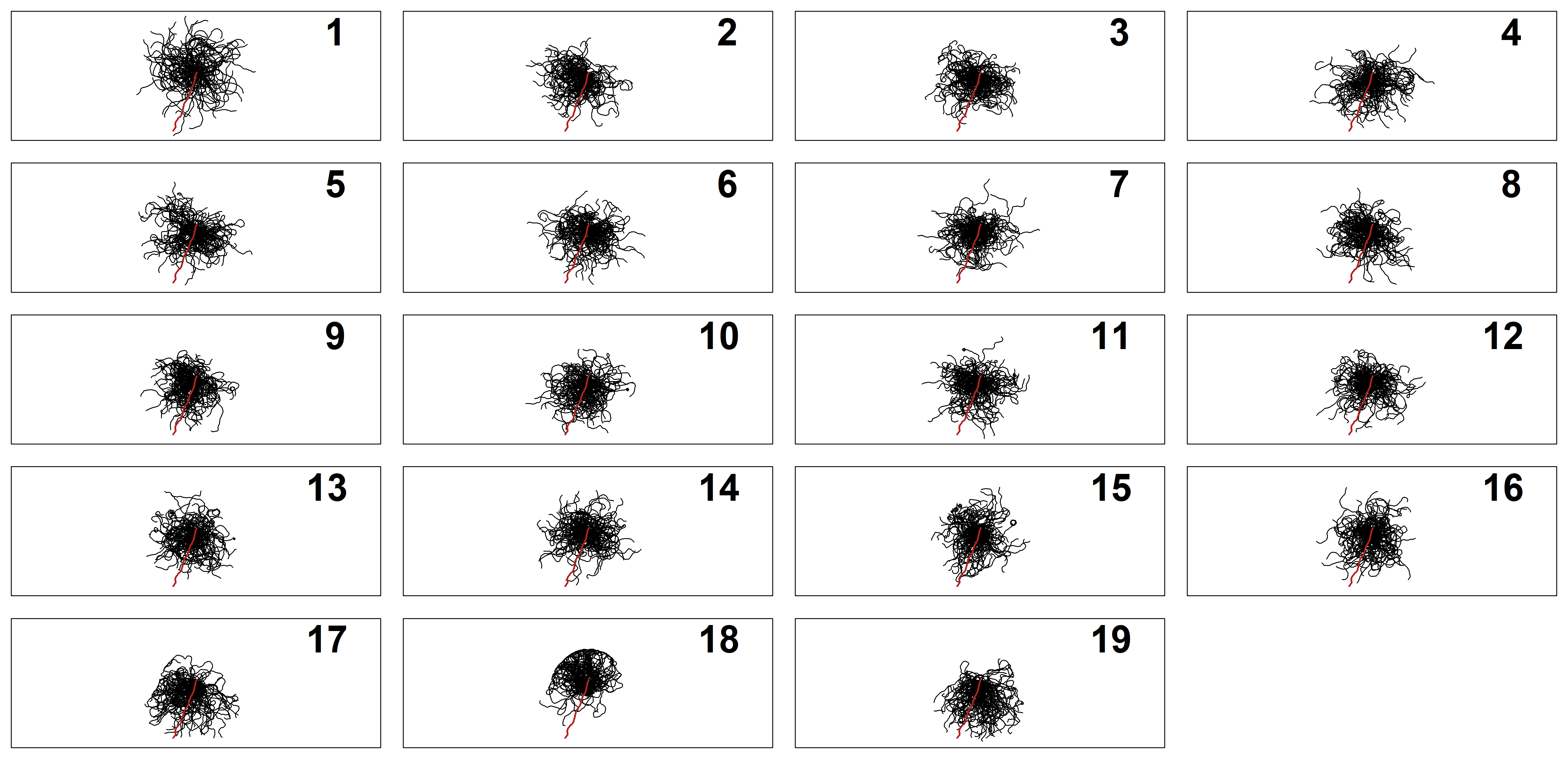


*Figure S5 The sub figures represent the output of the CRW models from 5 navigation strategies with different combinations of geomagnetic inputs (see Table S2 for reference) for animal 3. The labelling is as per the following: No bias (1), Constant heading (2 Max F, 3 Max H, 4 Max I, 5 Min F, 6 Min H, 7 Min I), Combination Bi-gradient taxis-Constant heading (8 FH, 9 FI, 10 HF, 11 HI, 12 IF, 13 IH), Bi-gradient taxis (14 FH, 15 FI, 16 IH), Geomagnetic taxis (17 F, 18 H, 19 I).*

Animal 4
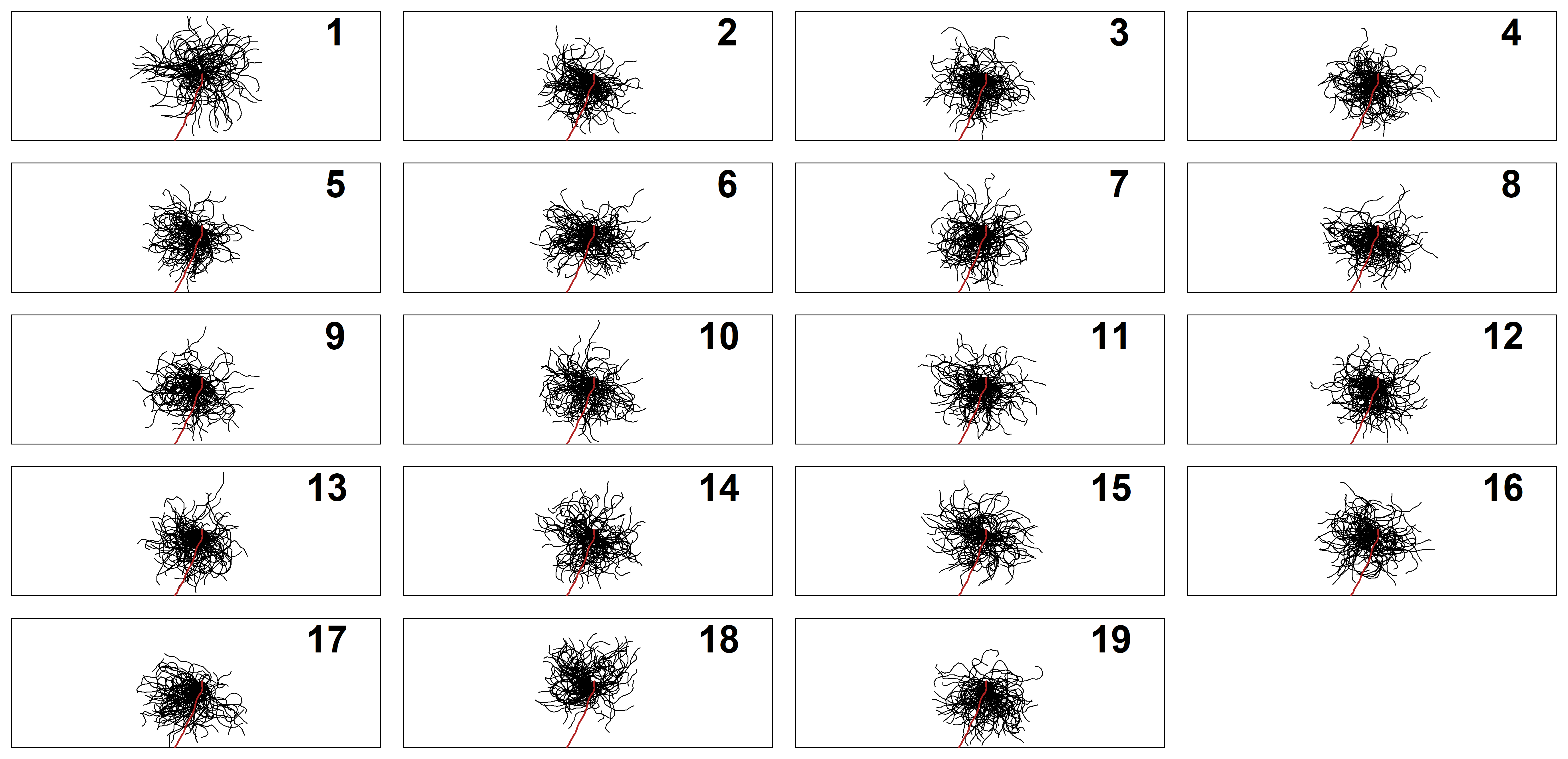


*Figure S6 The sub figures represent the output of the CRW models from 5 navigation strategies with different combinations of geomagnetic inputs (see Table S2 for reference) for animal 4. The labelling is as per the following: No bias (1), Constant heading (2 Max F, 3 Max H, 4 Max I, 5 Min F, 6 Min H, 7 Min I), Combination Bi-gradient taxis-Constant heading (8 FH, 9 FI, 10 HF, 11 HI, 12 IF, 13 IH), Bi-gradient taxis (14 FH, 15 FI, 16 IH), Geomagnetic taxis (17 F, 18 H, 19 I).*

Animal 5
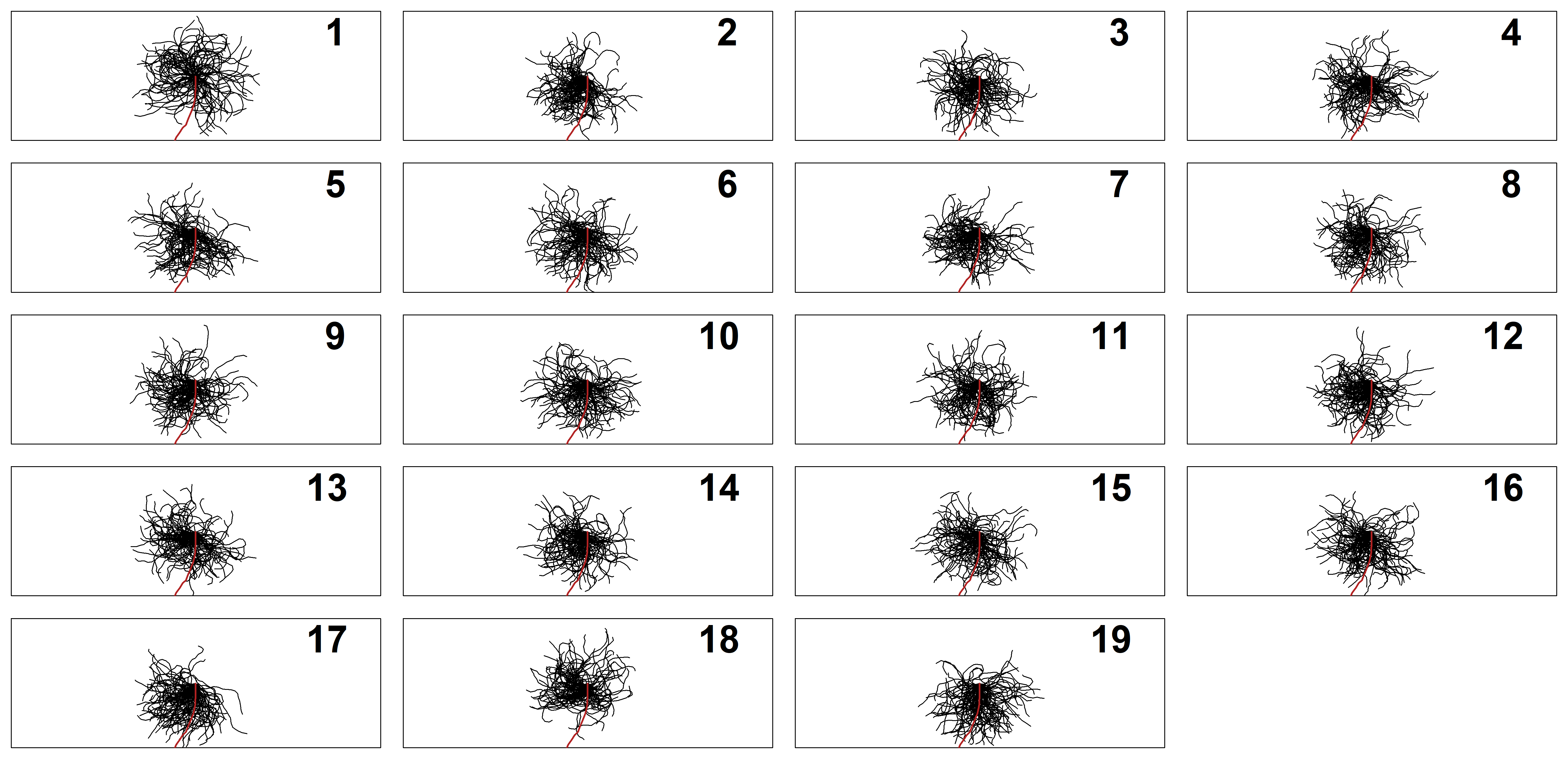


*Figure S7 The sub figures represent the output of the CRW models from 5 navigation strategies with different combinations of geomagnetic inputs (see Table S2 for reference) for animal 1. The labelling is as per the following: No bias (1), Constant heading (2 Max F, 3 Max H, 4 Max I, 5 Min F, 6 Min H, 7 Min I), Combination Bi-gradient taxis-Constant heading (8 FH, 9 FI, 10 HF, 11 HI, 12 IF, 13 IH), Bi-gradient taxis (14 FH, 15 FI, 16 IH), Geomagnetic taxis (17 F, 18 H, 19 I).*

Animal 6
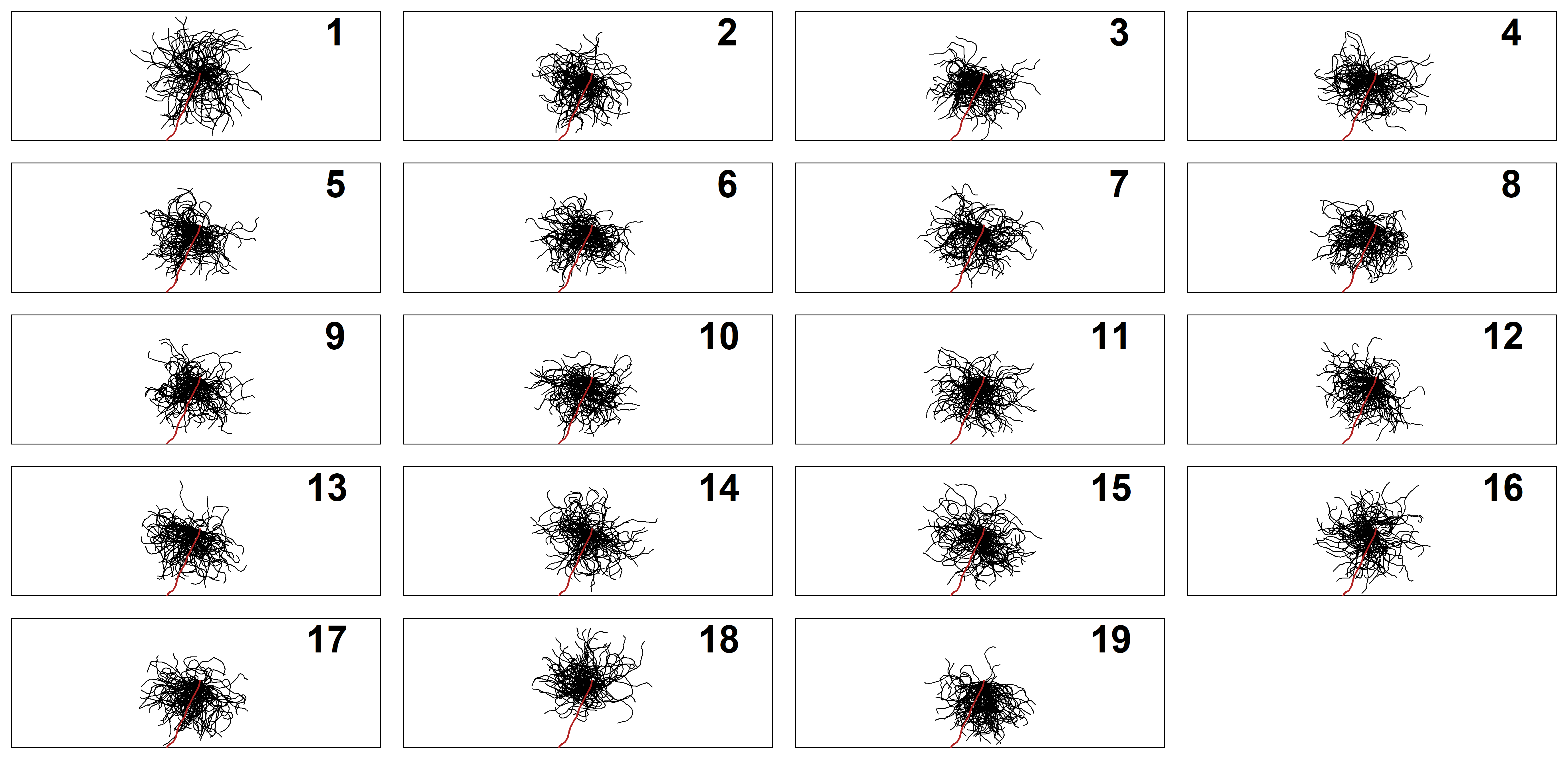


*Figure S8 The sub figures represent the output of the CRW models from 5 navigation strategies with different combinations of geomagnetic inputs (see Table S2 for reference) for animal 6. The labelling is as per the following: No bias (1), Constant heading (2 Max F, 3 Max H, 4 Max I, 5 Min F, 6 Min H, 7 Min I), Combination Bi-gradient taxis-Constant heading (8 FH, 9 FI, 10 HF, 11 HI, 12 IF, 13 IH), Bi-gradient taxis (14 FH, 15 FI, 16 IH), Geomagnetic taxis (17 F, 18 H, 19 I).*

Animal 7
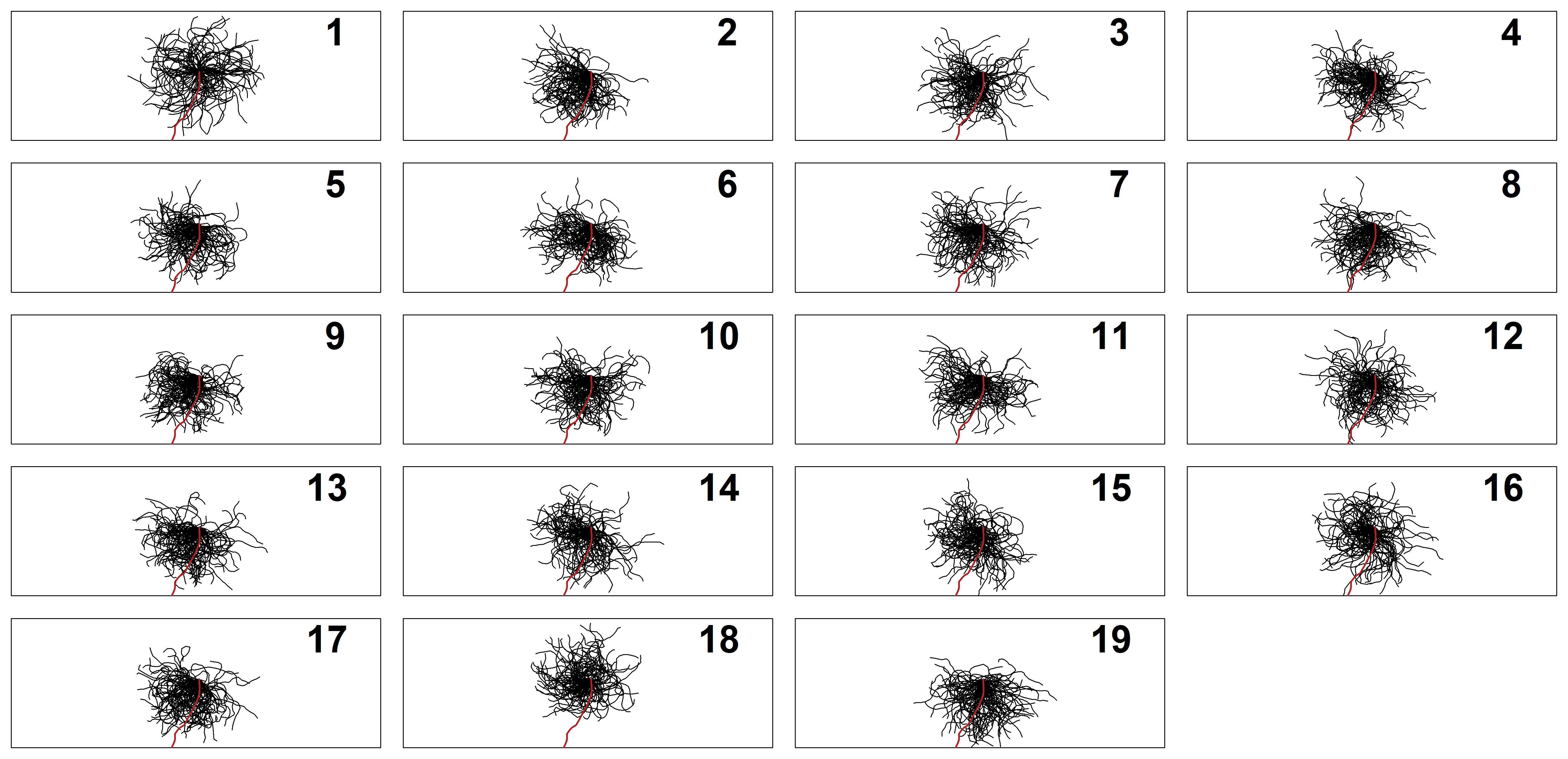


*Figure S9 The sub figures represent the output of the CRW models from 5 navigation strategies with different combinations of geomagnetic inputs (see Table S2 for reference) for animal 7. The labelling is as per the following: No bias (1), Constant heading (2 Max F, 3 Max H, 4 Max I, 5 Min F, 6 Min H, 7 Min I), Combination Bi-gradient taxis-Constant heading (8 FH, 9 FI, 10 HF, 11 HI, 12 IF, 13 IH), Bi-gradient taxis (14 FH, 15 FI, 16 IH), Geomagnetic taxis (17 F, 18 H, 19 I).*

Animal 8
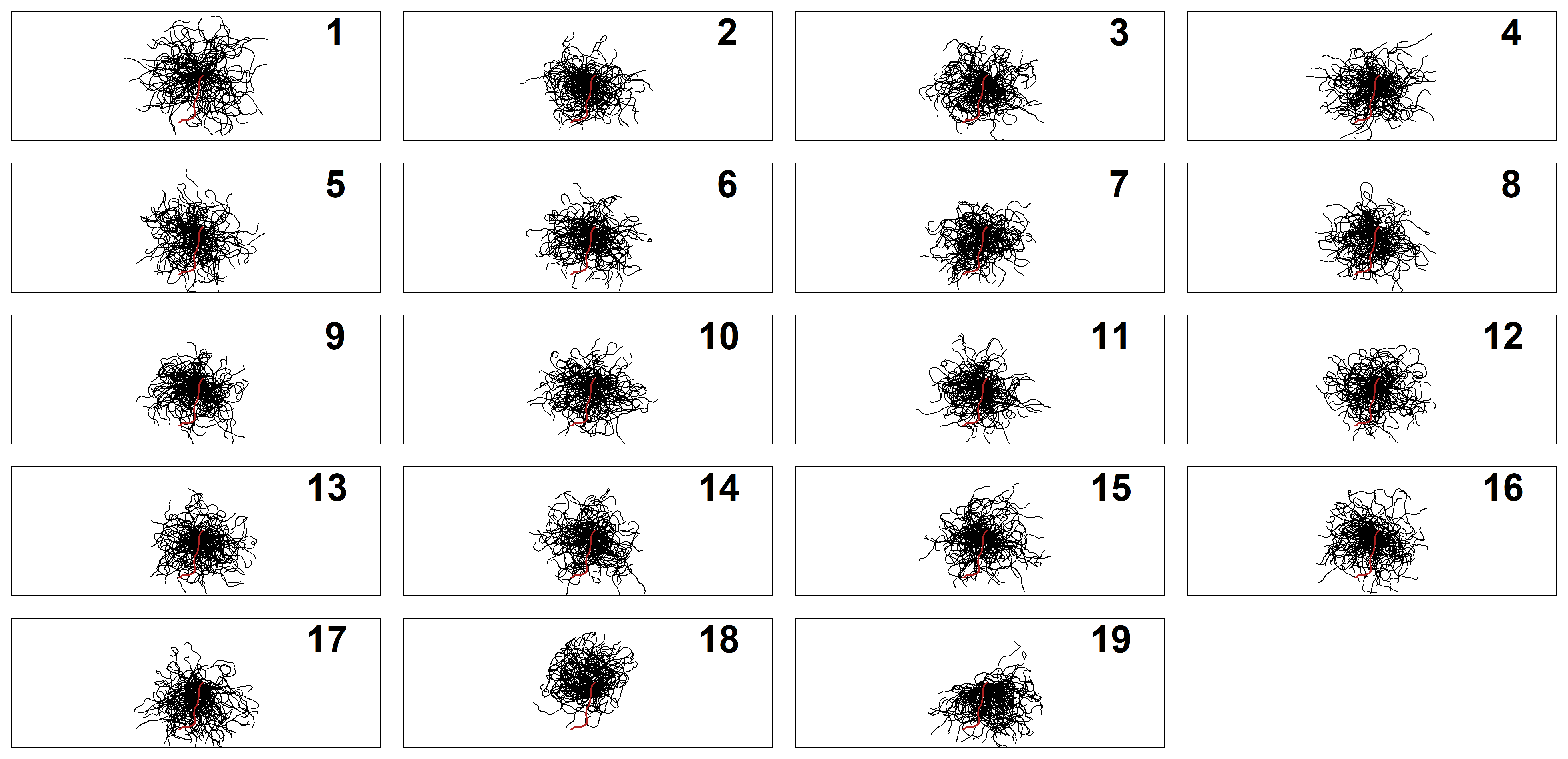


*Figure S10 The sub figures represent the output of the CRW models from 5 navigation strategies with different combinations of geomagnetic inputs (see Table S2 for reference) for animal 8. The labelling is as per the following: No bias (1), Constant heading (2 Max F, 3 Max H, 4 Max I, 5 Min F, 6 Min H, 7 Min I), Combination Bi-gradient taxis-Constant heading (8 FH, 9 FI, 10 HF, 11 HI, 12 IF, 13 IH), Bi-gradient taxis (14 FH, 15 FI, 16 IH), Geomagnetic taxis (17 F, 18 H, 19 I).*

Animal 9
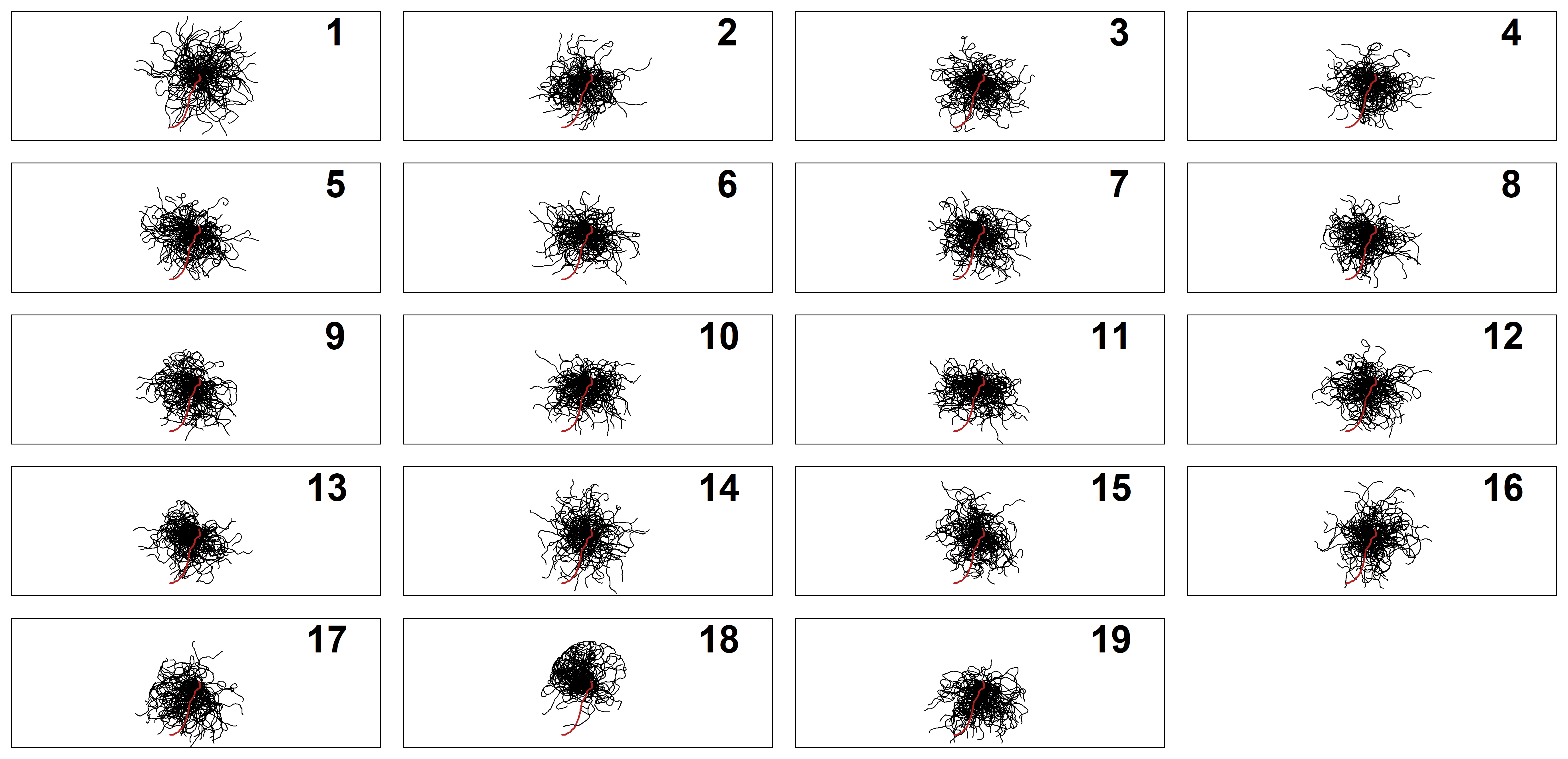


*Figure S11 The sub figures represent the output of the CRW models from 5 navigation strategies with different combinations of geomagnetic inputs (see Table S2 for reference) for animal 9. The labelling is as per the following: No bias (1), Constant heading (2 Max F, 3 Max H, 4 Max I, 5 Min F, 6 Min H, 7 Min I), Combination Bi-gradient taxis-Constant heading (8 FH, 9 FI, 10 HF, 11 HI, 12 IF, 13 IH), Bi-gradient taxis (14 FH, 15 FI, 16 IH), Geomagnetic taxis (17 F, 18 H, 19 I).*

Animal 10
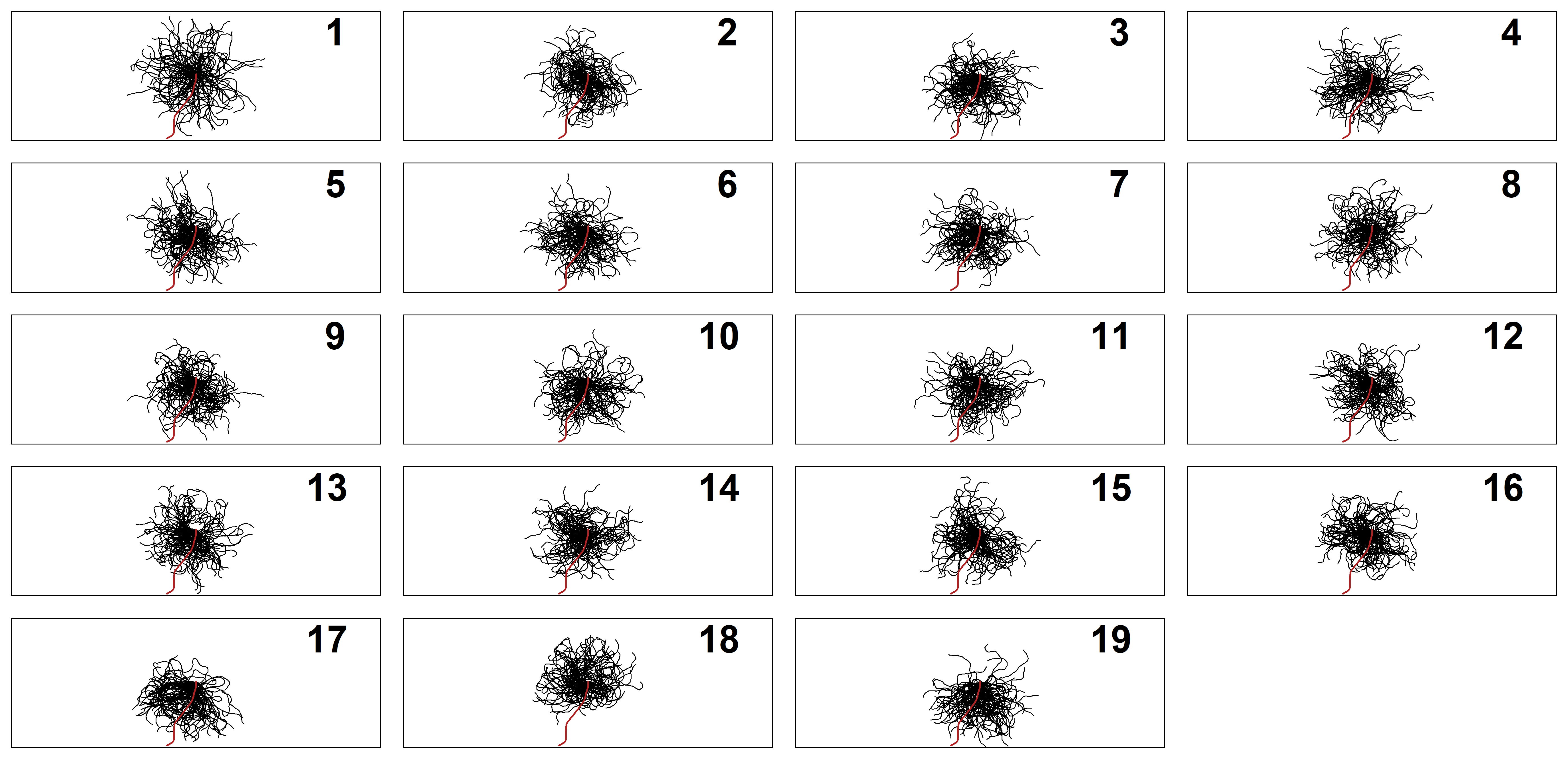


*Figure S12 The sub figures represent the output of the CRW models from 5 navigation strategies with different combinations of geomagnetic inputs (see Table S2 for reference) for animal 10. The labelling is as per the following: No bias (1), Constant heading (2 Max F, 3 Max H, 4 Max I, 5 Min F, 6 Min H, 7 Min I), Combination Bi-gradient taxis-Constant heading (8 FH, 9 FI, 10 HF, 11 HI, 12 IF, 13 IH), Bi-gradient taxis (14 FH, 15 FI, 16 IH), Geomagnetic taxis (17 F, 18 H, 19 I).*

Animal 11
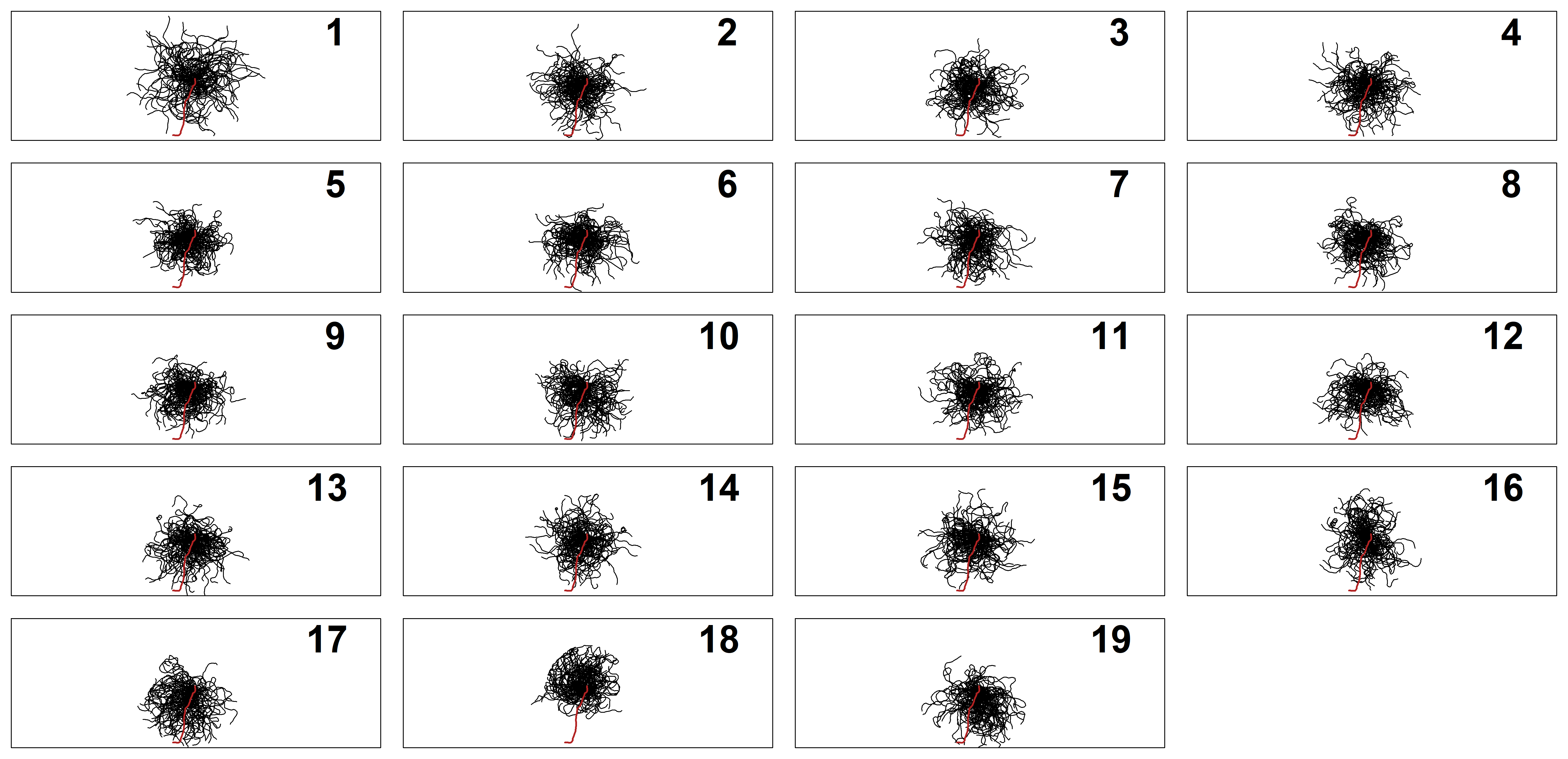


*Figure S13 The sub figures represent the output of the CRW models from 5 navigation strategies with different combinations of geomagnetic inputs (see Table S2 for reference) for animal 11. The labelling is as per the following: No bias (1), Constant heading (2 Max F, 3 Max H, 4 Max I, 5 Min F, 6 Min H, 7 Min I), Combination Bi-gradient taxis-Constant heading (8 FH, 9 FI, 10 HF, 11 HI, 12 IF, 13 IH), Bi-gradient taxis (14 FH, 15 FI, 16 IH), Geomagnetic taxis (17 F, 18 H, 19 I).*

Animal 12
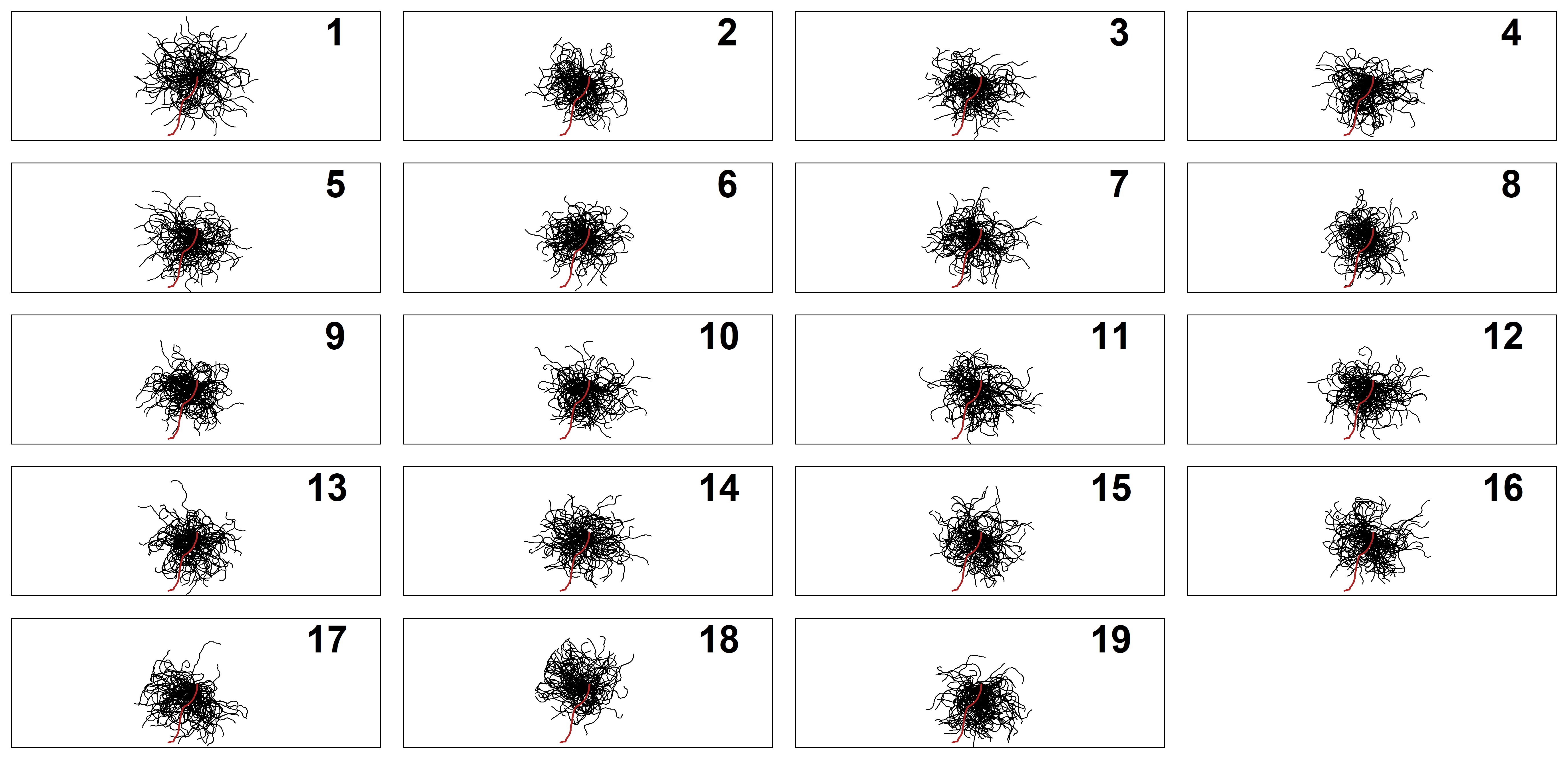


*Figure S14 The sub figures represent the output of the CRW models from 5 navigation strategies with different combinations of geomagnetic inputs (see Table S2 for reference) for animal 12. The labelling is as per the following: No bias (1), Constant heading (2 Max F, 3 Max H, 4 Max I, 5 Min F, 6 Min H, 7 Min I), Combination Bi-gradient taxis-Constant heading (8 FH, 9 FI, 10 HF, 11 HI, 12 IF, 13 IH), Bi-gradient taxis (14 FH, 15 FI, 16 IH), Geomagnetic taxis (17 F, 18 H, 19 I).*

Animal 13
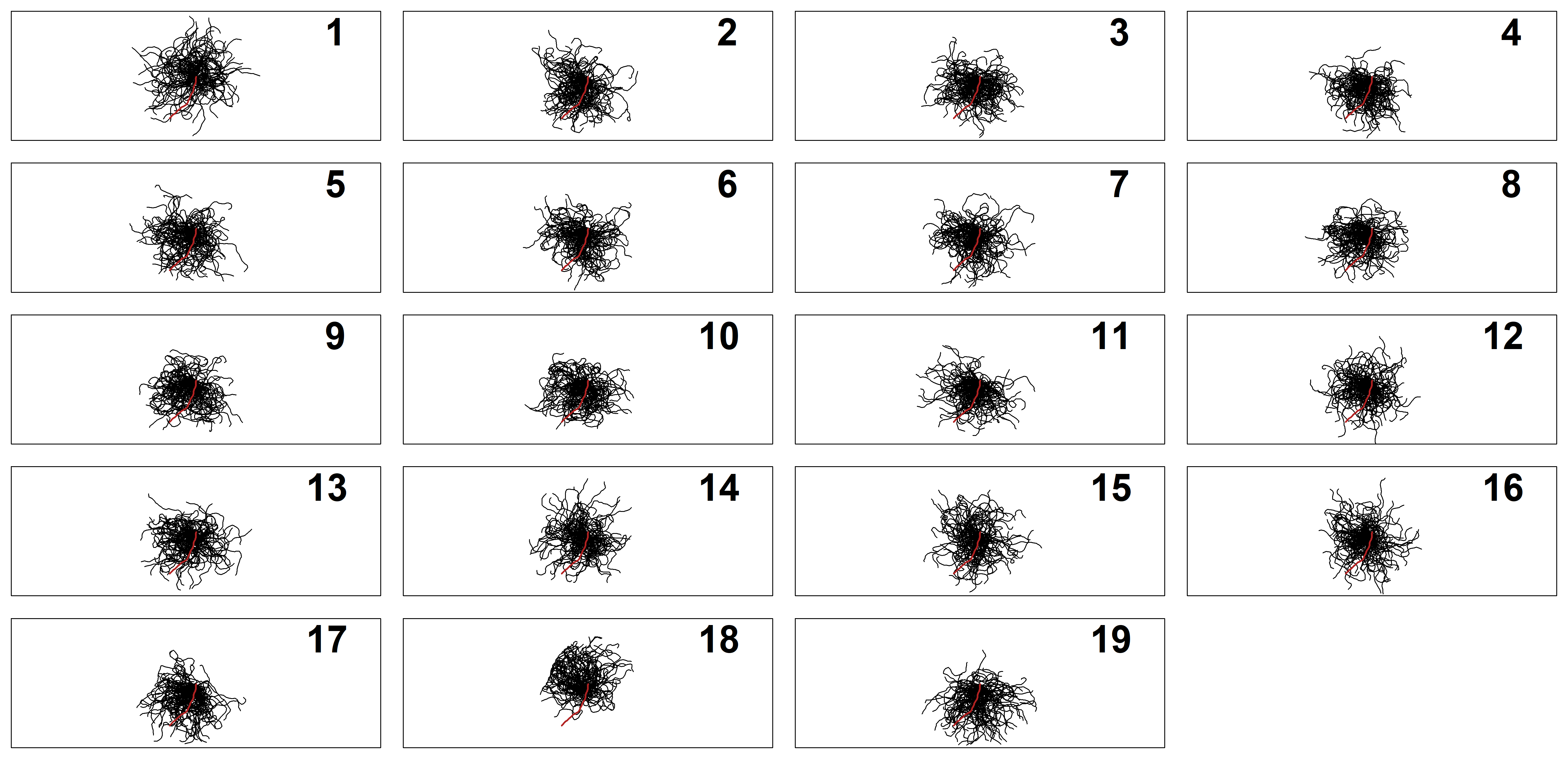


*Figure S15 The sub figures represent the output of the CRW models from 5 navigation strategies with different combinations of geomagnetic inputs (see Table S2 for reference) for animal 13. The labelling is as per the following: No bias (1), Constant heading (2 Max F, 3 Max H, 4 Max I, 5 Min F, 6 Min H, 7 Min I), Combination Bi-gradient taxis-Constant heading (8 FH, 9 FI, 10 HF, 11 HI, 12 IF, 13 IH), Bi-gradient taxis (14 FH, 15 FI, 16 IH), Geomagnetic taxis (17 F, 18 H, 19 I).*

Animal 14
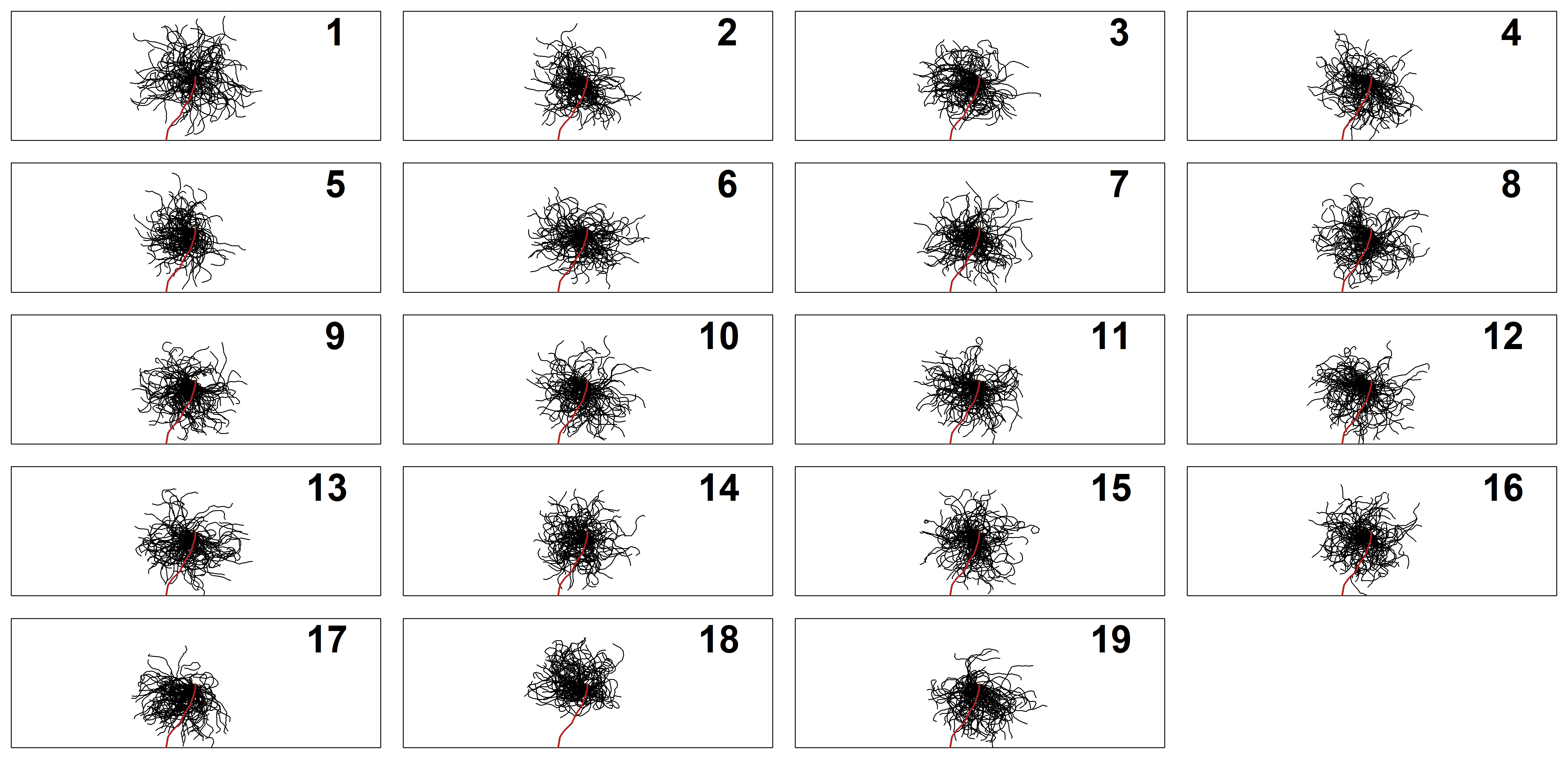


*Figure S16 The sub figures represent the output of the CRW models from 5 navigation strategies with different combinations of geomagnetic inputs (see Table S2 for reference) for animal 14. The labelling is as per the following: No bias (1), Constant heading (2 Max F, 3 Max H, 4 Max I, 5 Min F, 6 Min H, 7 Min I), Combination Bi-gradient taxis-Constant heading (8 FH, 9 FI, 10 HF, 11 HI, 12 IF, 13 IH), Bi-gradient taxis (14 FH, 15 FI, 16 IH), Geomagnetic taxis (17 F, 18 H, 19 I).*

**CRB**

Animal 1


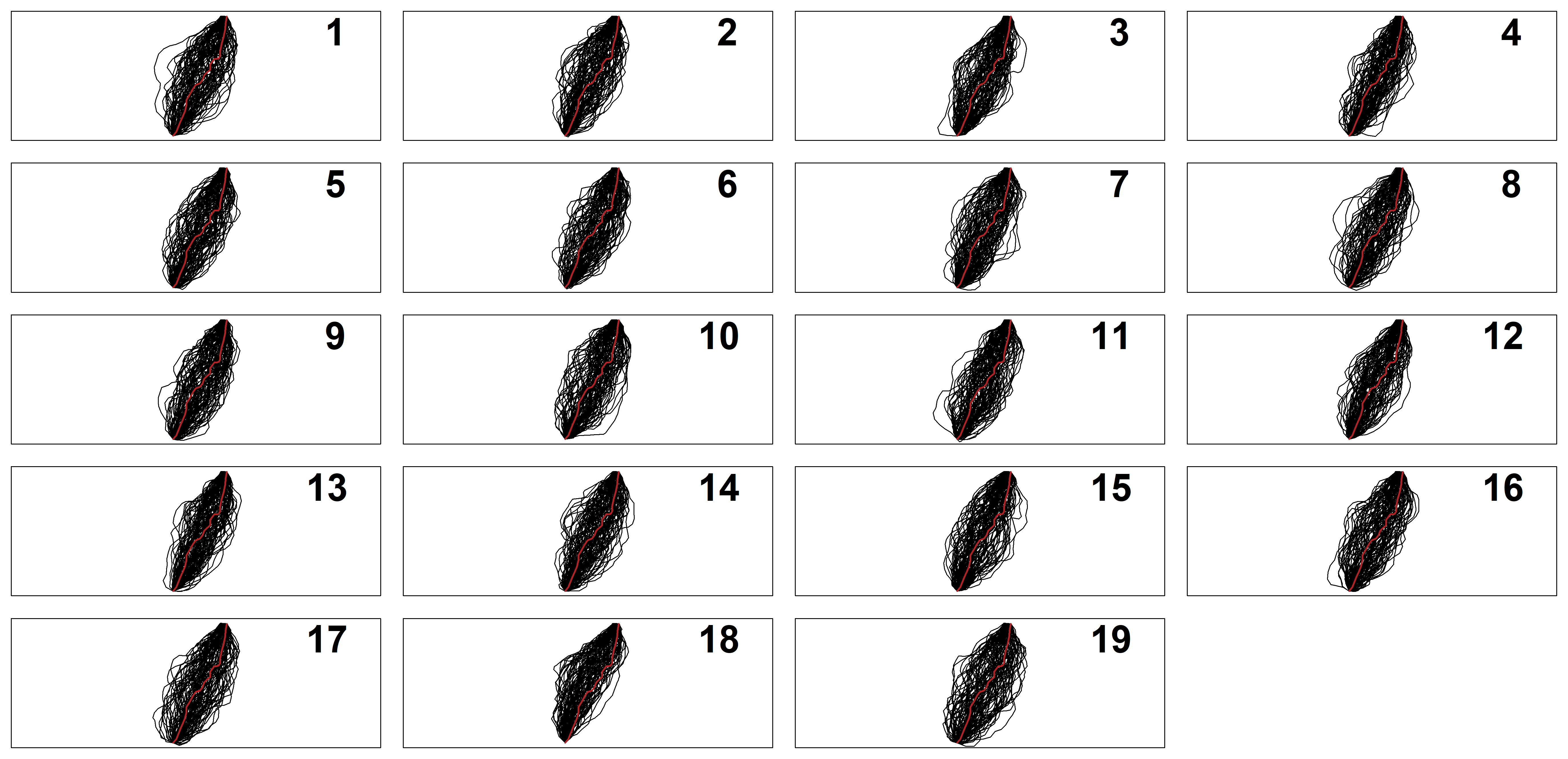


*Figure S17 The sub figures represent the output of the CRB models from 5 navigation strategies with different combinations of geomagnetic inputs (see Table S2 for reference) for animal 1. The labelling is as per the following: No bias (1), Constant heading (2 Max F, 3 Max H, 4 Max I, 5 Min F, 6 Min H, 7 Min I), Combination Bi-gradient taxis-Constant heading (8 FH, 9 FI, 10 HF, 11 HI, 12 IF, 13 IH), Bi-gradient taxis (14 FH, 15 FI, 16 IH), Geomagnetic taxis (17 F, 18 H, 19 I).*

Animal 2
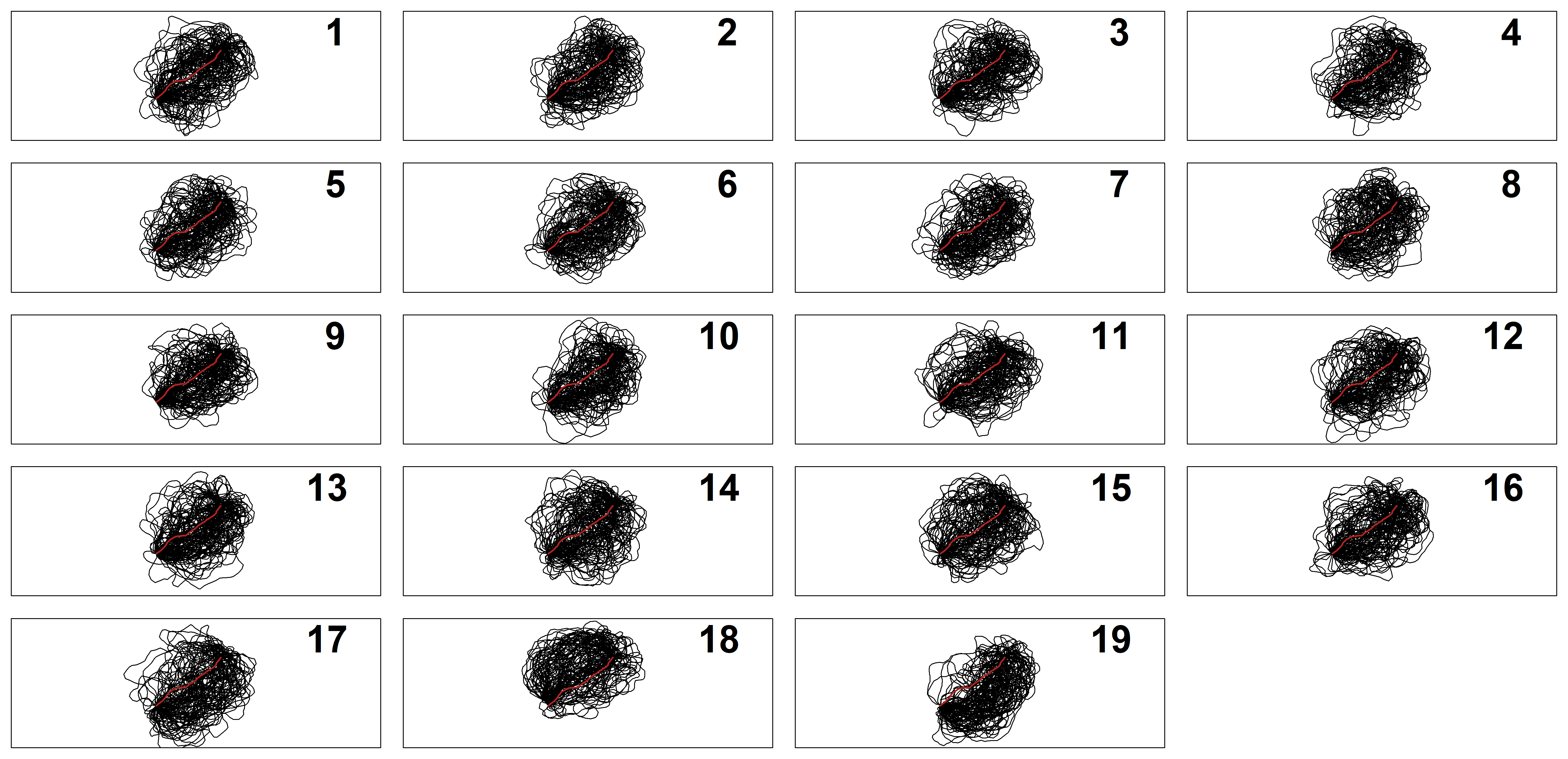


*Figure S18 The sub figures represent the output of the CRB models from 5 navigation strategies with different combinations of geomagnetic inputs (see Table S2 for reference) for animal 2. The labelling is as per the following: No bias (1), Constant heading (2 Max F, 3 Max H, 4 Max I, 5 Min F, 6 Min H, 7 Min I), Combination Bi-gradient taxis-Constant heading (8 FH, 9 FI, 10 HF, 11 HI, 12 IF, 13 IH), Bi-gradient taxis (14 FH, 15 FI, 16 IH), Geomagnetic taxis (17 F, 18 H, 19 I).*

Animal 3
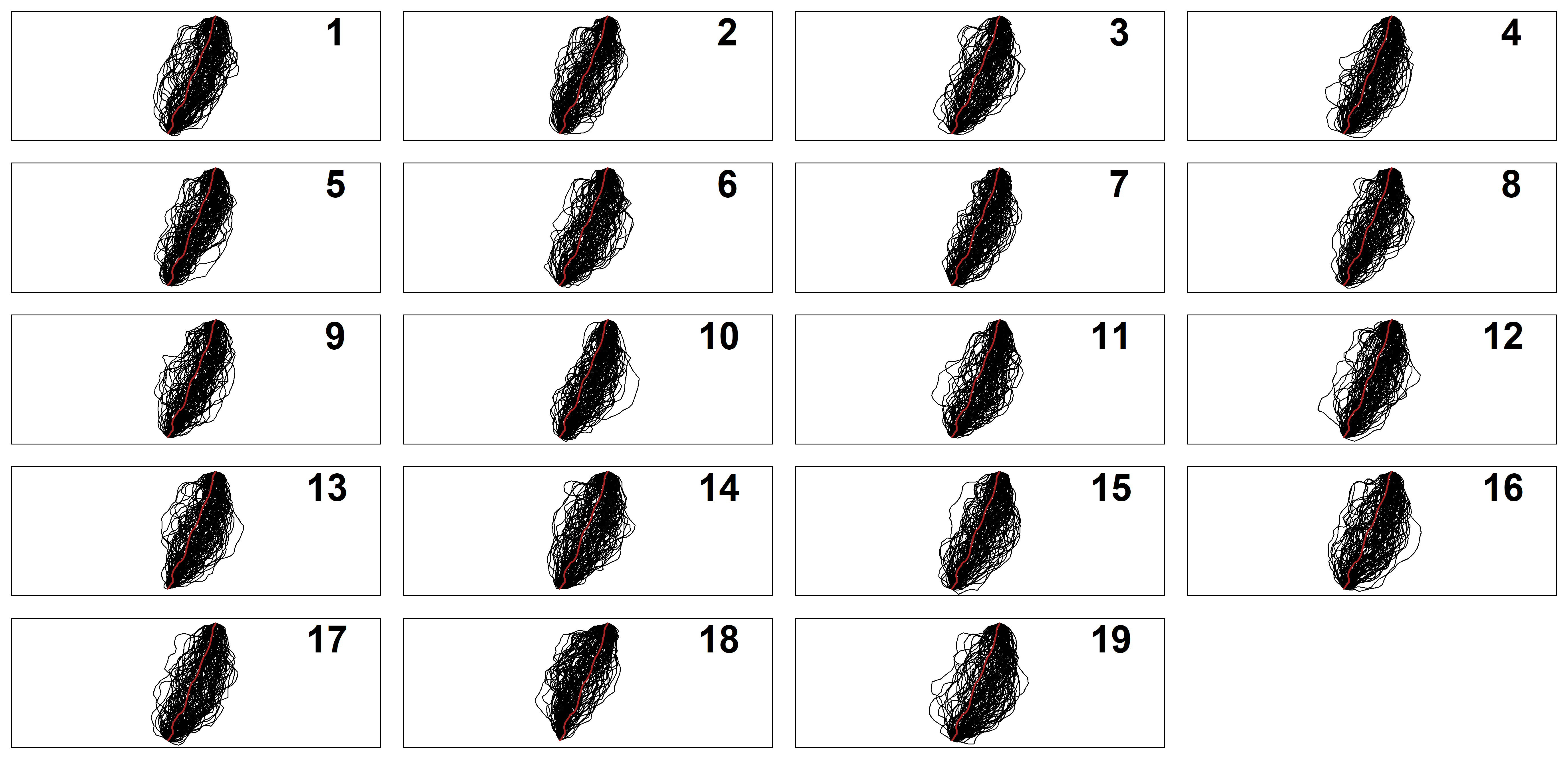


*Figure S19 The sub figures represent the output of the CRB models from 5 navigation strategies with different combinations of geomagnetic inputs (see Table S2 for reference) for animal 3. The labelling is as per the following: No bias (1), Constant heading (2 Max F, 3 Max H, 4 Max I, 5 Min F, 6 Min H, 7 Min I), Combination Bi-gradient taxis-Constant heading (8 FH, 9 FI, 10 HF, 11 HI, 12 IF, 13 IH), Bi-gradient taxis (14 FH, 15 FI, 16 IH), Geomagnetic taxis (17 F, 18 H, 19 I).*

Animal 4
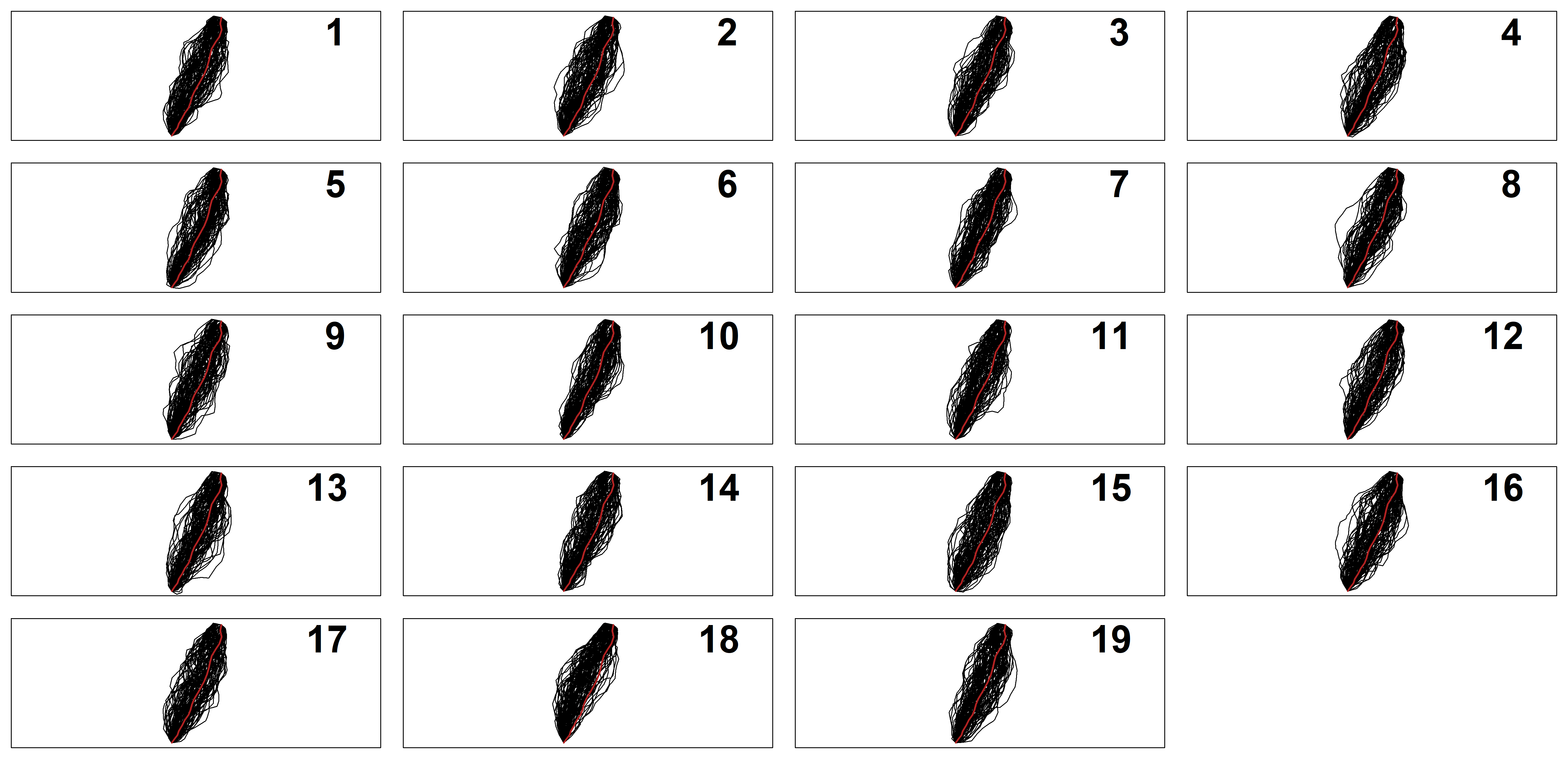


*Figure S20 The sub figures represent the output of the CRB models from 5 navigation strategies with different combinations of geomagnetic inputs (see Table S2 for reference) for animal 4. The labelling is as per the following: No bias (1), Constant heading (2 Max F, 3 Max H, 4 Max I, 5 Min F, 6 Min H, 7 Min I), Combination Bi-gradient taxis-Constant heading (8 FH, 9 FI, 10 HF, 11 HI, 12 IF, 13 IH), Bi-gradient taxis (14 FH, 15 FI, 16 IH), Geomagnetic taxis (17 F, 18 H, 19 I).*

Animal 5
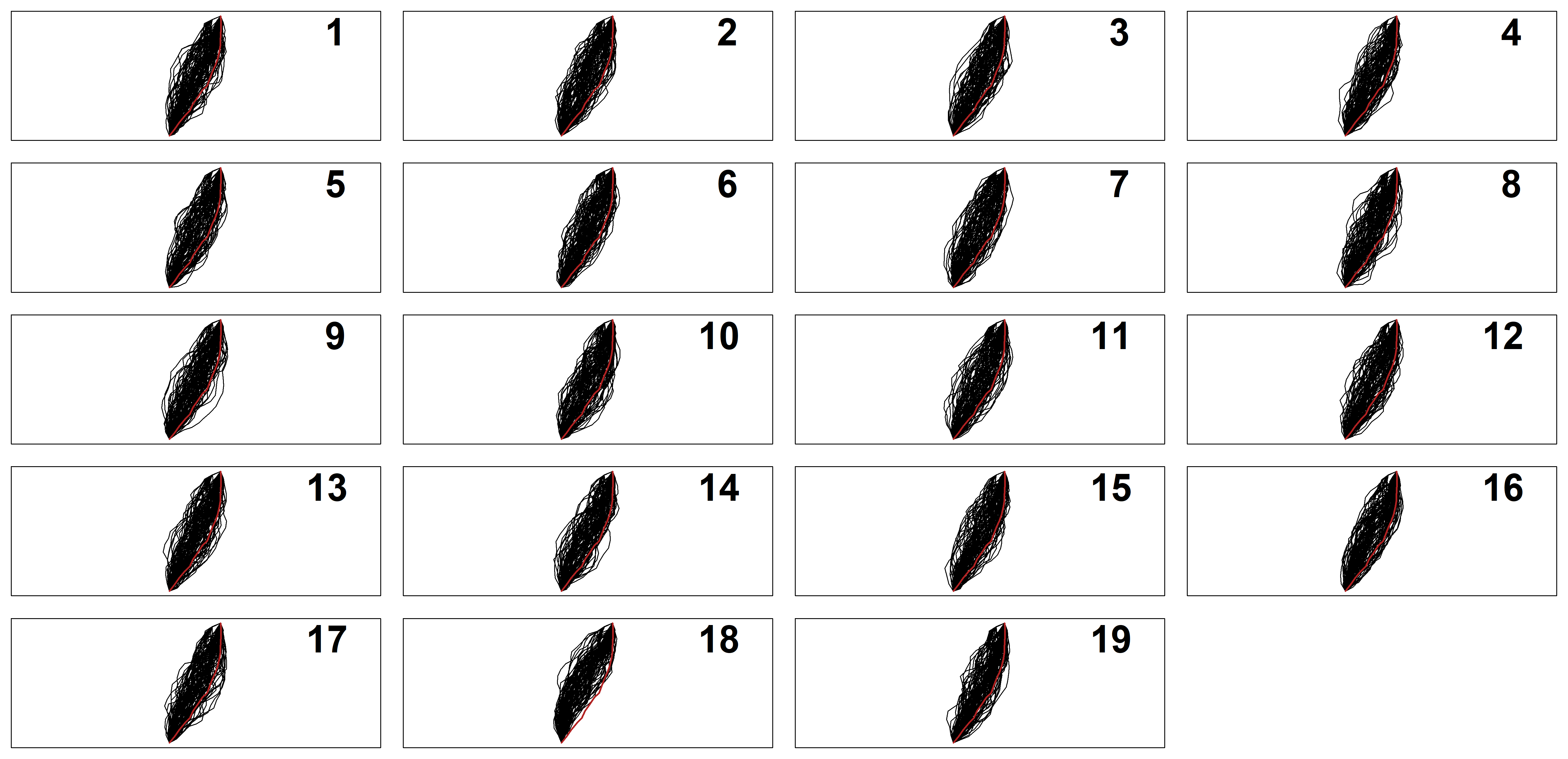


*Figure S21 The sub figures represent the output of the CRB models from 5 navigation strategies with different combinations of geomagnetic inputs (see Table S2 for reference) for animal 5. The labelling is as per the following: No bias (1), Constant heading (2 Max F, 3 Max H, 4 Max I, 5 Min F, 6 Min H, 7 Min I), Combination Bi-gradient taxis-Constant heading (8 FH, 9 FI, 10 HF, 11 HI, 12 IF, 13 IH), Bi-gradient taxis (14 FH, 15 FI, 16 IH), Geomagnetic taxis (17 F, 18 H, 19 I).*

Animal 6
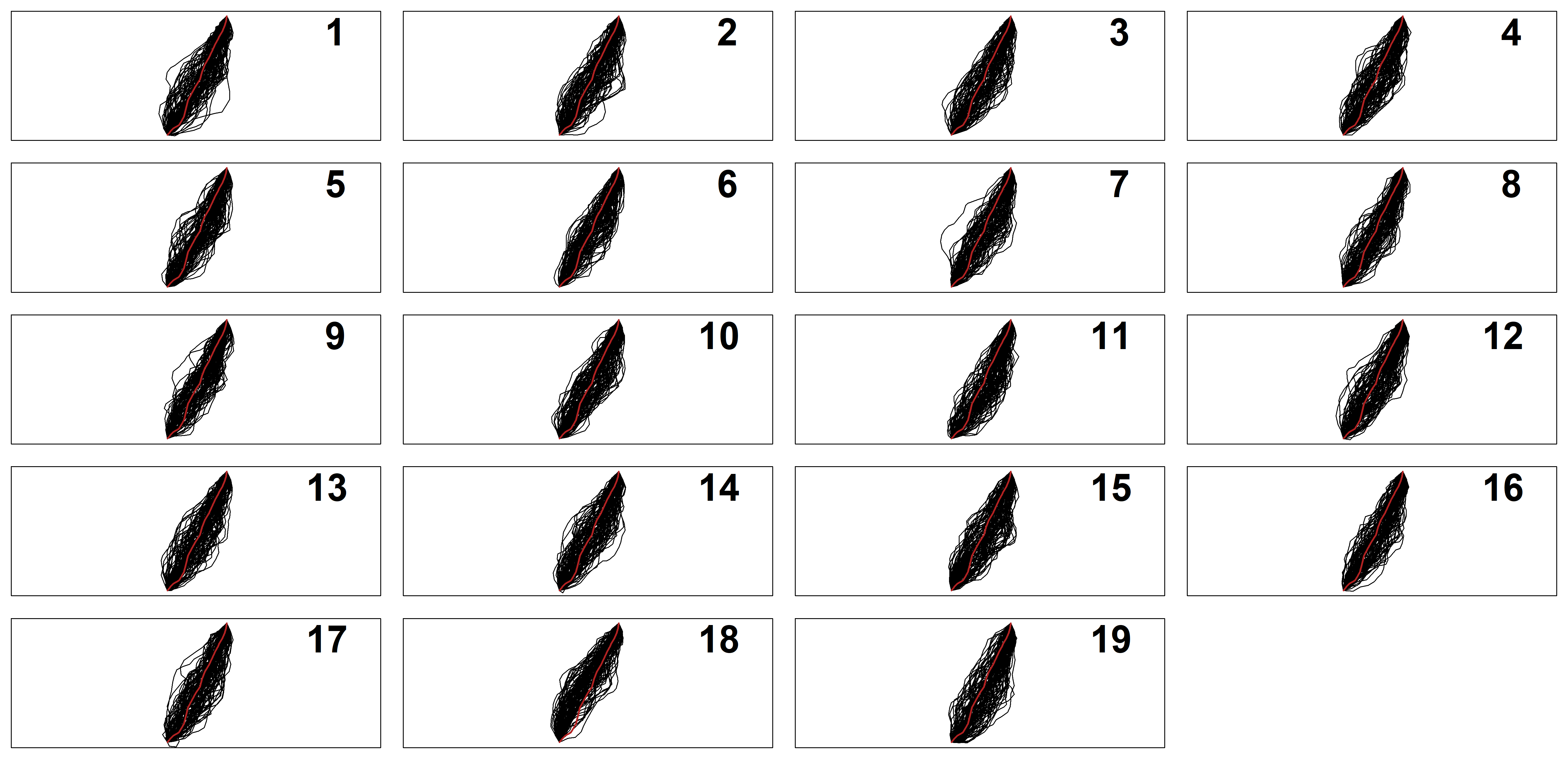


*Figure S22 The sub figures represent the output of the CRB models from 5 navigation strategies with different combinations of geomagnetic inputs (see Table S2 for reference) for animal 6. The labelling is as per the following: No bias (1), Constant heading (2 Max F, 3 Max H, 4 Max I, 5 Min F, 6 Min H, 7 Min I), Combination Bi-gradient taxis-Constant heading (8 FH, 9 FI, 10 HF, 11 HI, 12 IF, 13 IH), Bi-gradient taxis (14 FH, 15 FI, 16 IH), Geomagnetic taxis (17 F, 18 H, 19 I).*

Animal 7
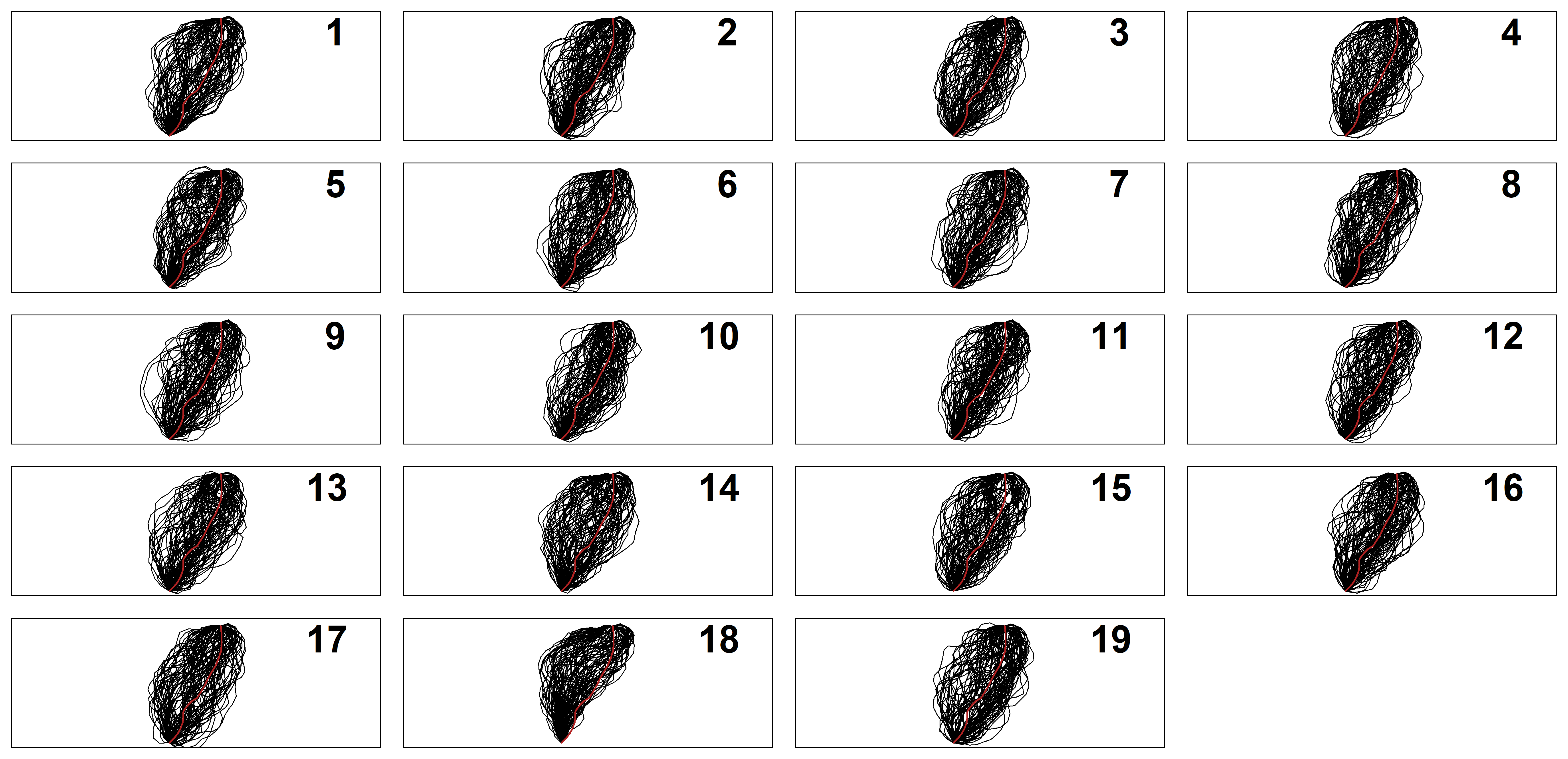


*Figure S23 The sub figures represent the output of the CRB models from 5 navigation strategies with different combinations of geomagnetic inputs (see Table S2 for reference) for animal 7. The labelling is as per the following: No bias (1), Constant heading (2 Max F, 3 Max H, 4 Max I, 5 Min F, 6 Min H, 7 Min I), Combination Bi-gradient taxis-Constant heading (8 FH, 9 FI, 10 HF, 11 HI, 12 IF, 13 IH), Bi-gradient taxis (14 FH, 15 FI, 16 IH), Geomagnetic taxis (17 F, 18 H, 19 I).*

Animal 8
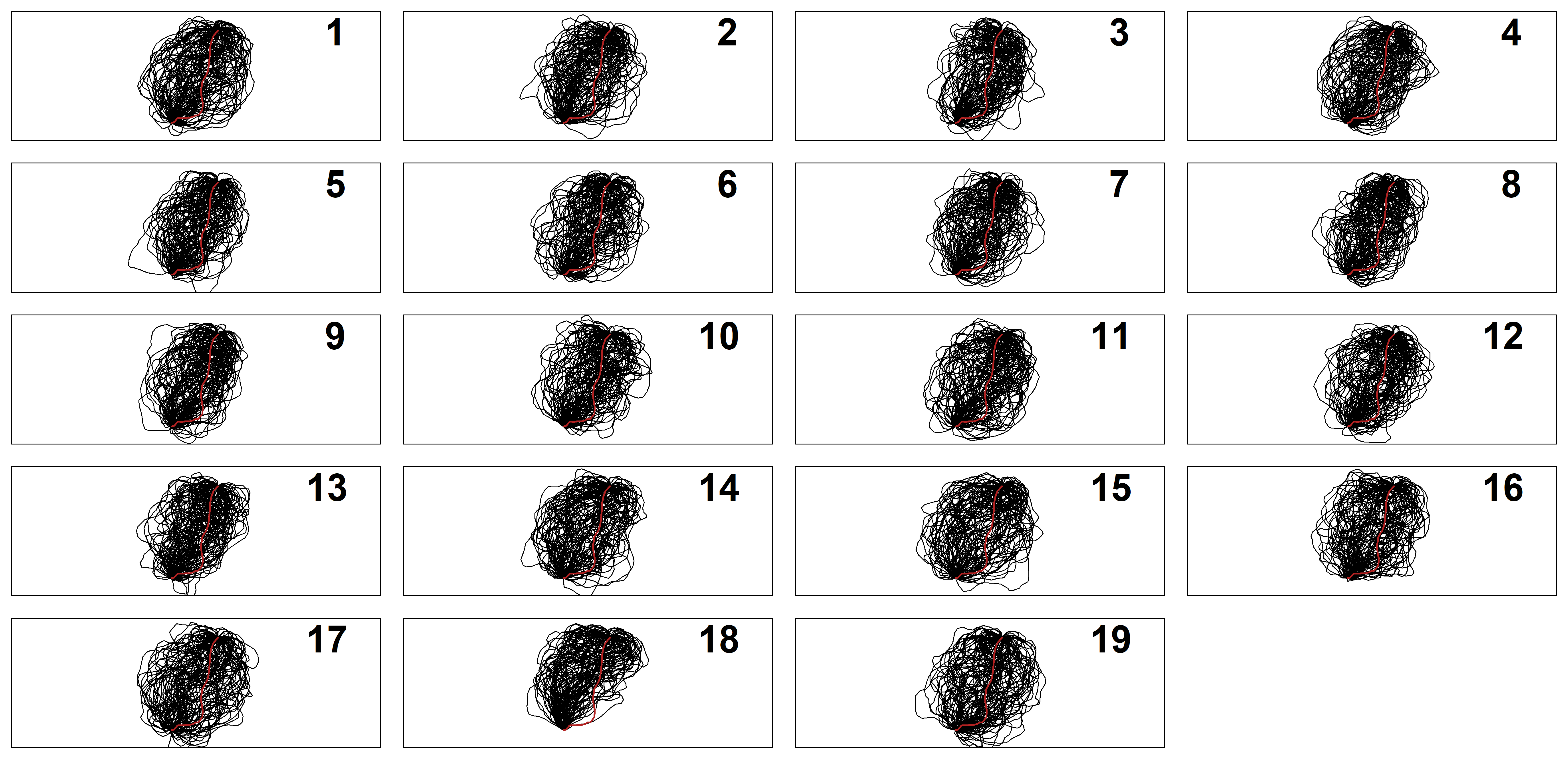


*Figure S24 The sub figures represent the output of the CRB models from 5 navigation strategies with different combinations of geomagnetic inputs (see Table S2 for reference) for animal 8. The labelling is as per the following: No bias (1), Constant heading (2 Max F, 3 Max H, 4 Max I, 5 Min F, 6 Min H, 7 Min I), Combination Bi-gradient taxis-Constant heading (8 FH, 9 FI, 10 HF, 11 HI, 12 IF, 13 IH), Bi-gradient taxis (14 FH, 15 FI, 16 IH), Geomagnetic taxis (17 F, 18 H, 19 I).*

Animal 9
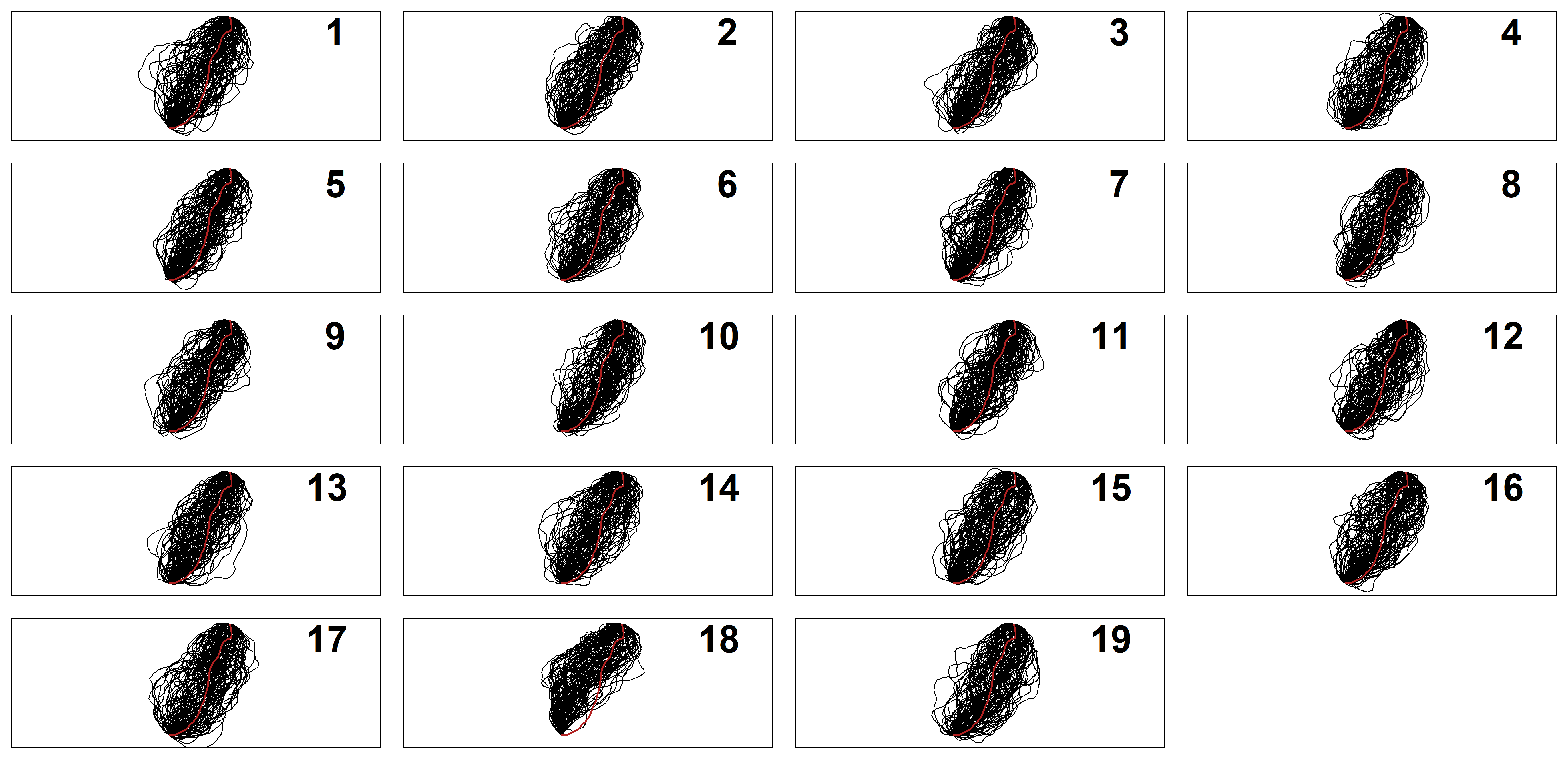


*Figure S25 The sub figures represent the output of the CRB models from 5 navigation strategies with different combinations of geomagnetic inputs (see Table S2 for reference) for animal 9. The labelling is as per the following: No bias (1), Constant heading (2 Max F, 3 Max H, 4 Max I, 5 Min F, 6 Min H, 7 Min I), Combination Bi-gradient taxis-Constant heading (8 FH, 9 FI, 10 HF, 11 HI, 12 IF, 13 IH), Bi-gradient taxis (14 FH, 15 FI, 16 IH), Geomagnetic taxis (17 F, 18 H, 19 I).*

Animal 10
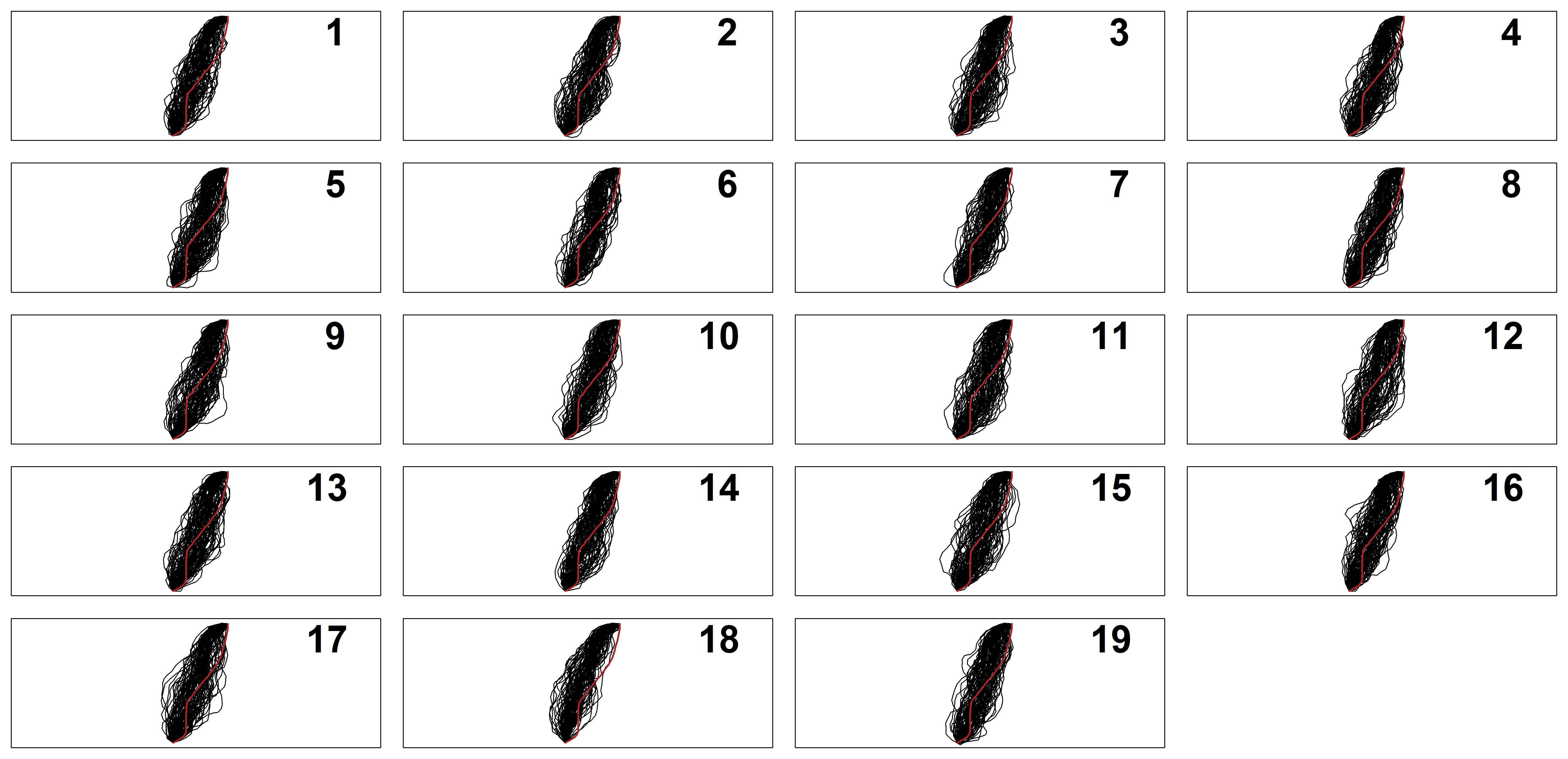


*Figure S26 The sub figures represent the output of the CRB models from 5 navigation strategies with different combinations of geomagnetic inputs (see Table S2 for reference) for animal 10. The labelling is as per the following: No bias (1), Constant heading (2 Max F, 3 Max H, 4 Max I, 5 Min F, 6 Min H, 7 Min I), Combination Bi-gradient taxis-Constant heading (8 FH, 9 FI, 10 HF, 11 HI, 12 IF, 13 IH), Bi-gradient taxis (14 FH, 15 FI, 16 IH), Geomagnetic taxis (17 F, 18 H, 19 I).*

Animal 11
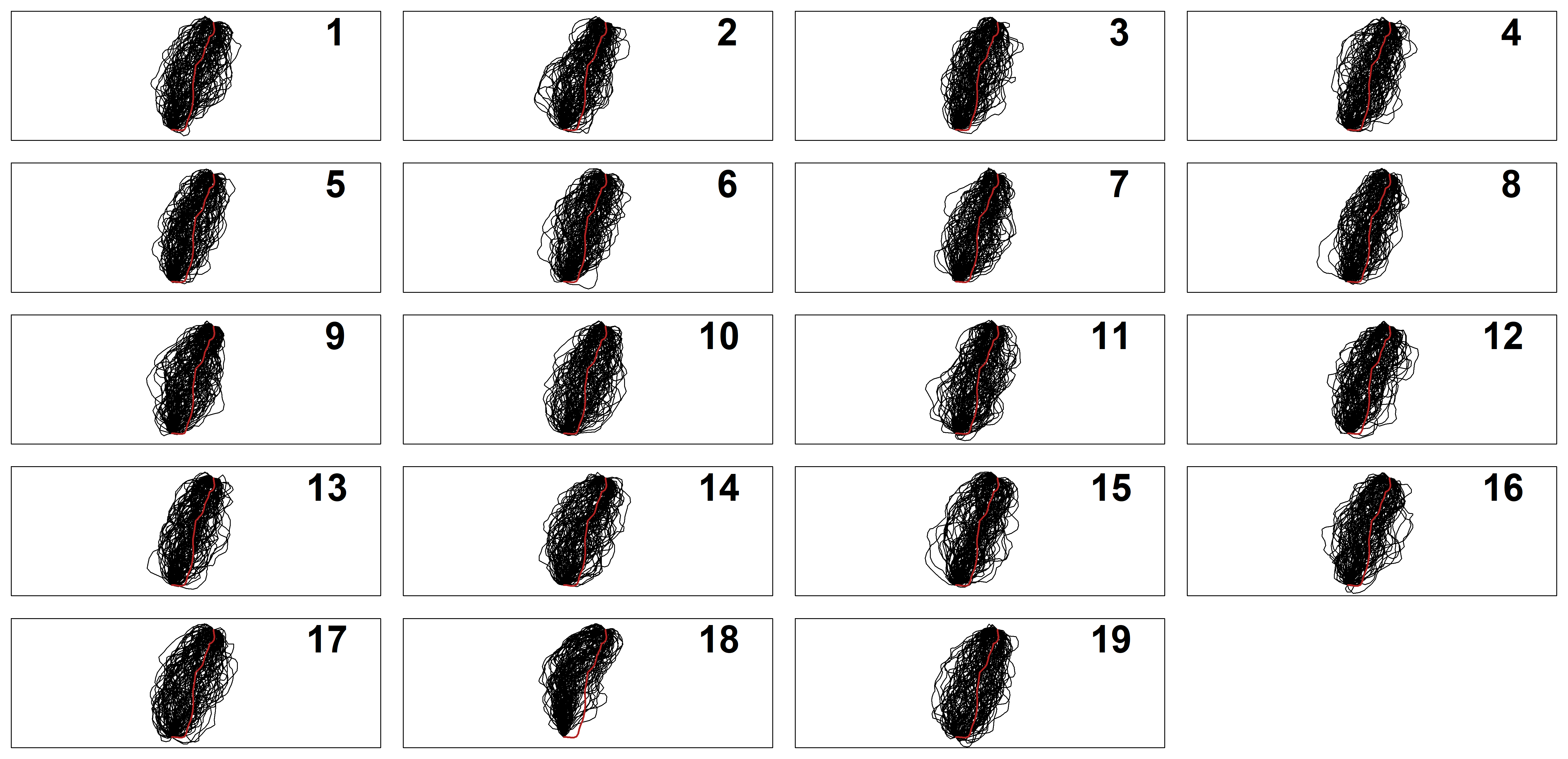


*Figure S27 The sub figures represent the output of the CRB models from 5 navigation strategies with different combinations of geomagnetic inputs (see Table S2 for reference) for animal 11. The labelling is as per the following: No bias (1), Constant heading (2 Max F, 3 Max H, 4 Max I, 5 Min F, 6 Min H, 7 Min I), Combination Bi-gradient taxis-Constant heading (8 FH, 9 FI, 10 HF, 11 HI, 12 IF, 13 IH), Bi-gradient taxis (14 FH, 15 FI, 16 IH), Geomagnetic taxis (17 F, 18 H, 19 I).*

Animal 12
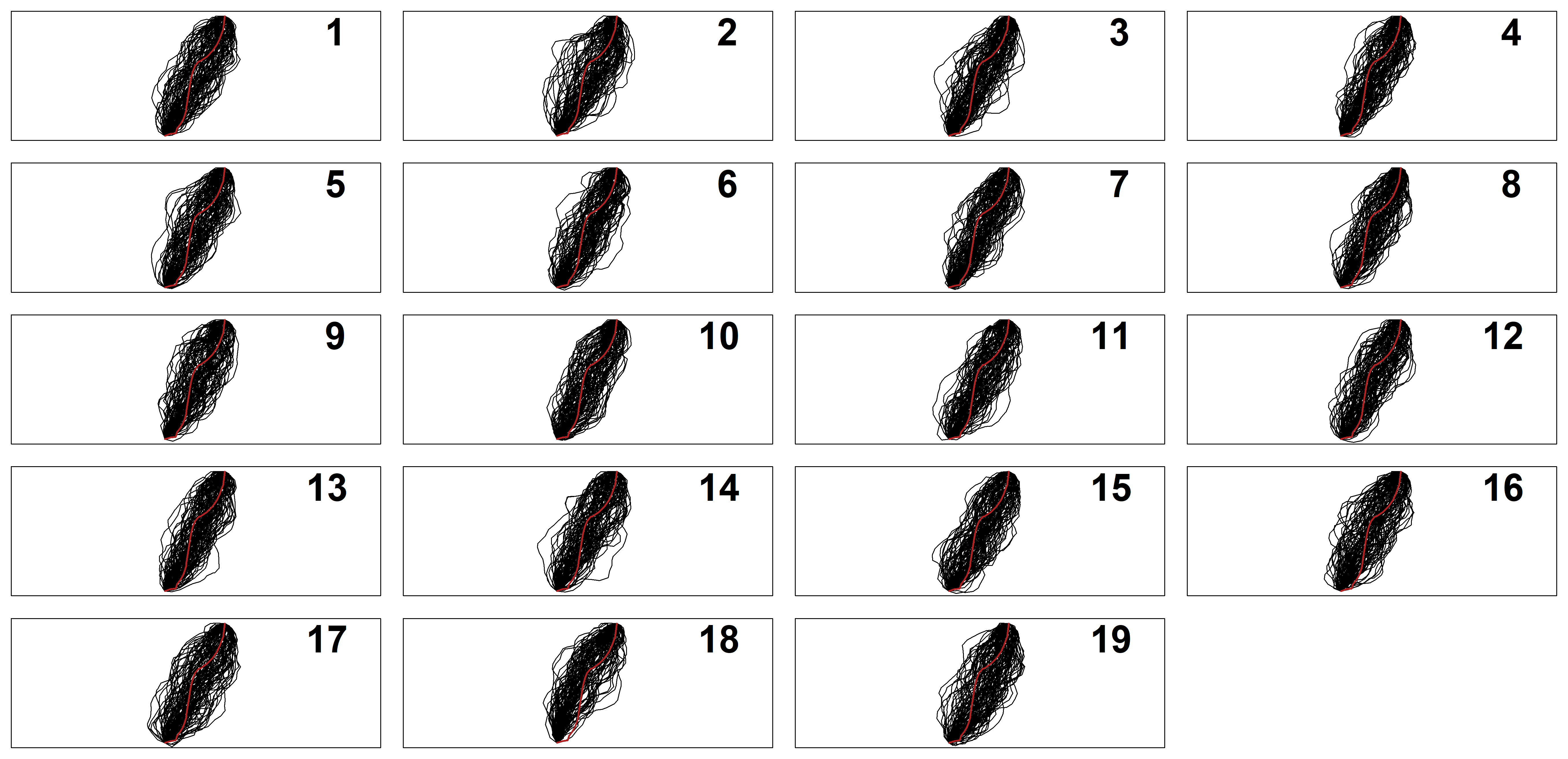


*Figure S28 The sub figures represent the output of the CRB models from 5 navigation strategies with different combinations of geomagnetic inputs (see Table S2 for reference) for animal 12. The labelling is as per the following: No bias (1), Constant heading (2 Max F, 3 Max H, 4 Max I, 5 Min F, 6 Min H, 7 Min I), Combination Bi-gradient taxis-Constant heading (8 FH, 9 FI, 10 HF, 11 HI, 12 IF, 13 IH), Bi-gradient taxis (14 FH, 15 FI, 16 IH), Geomagnetic taxis (17 F, 18 H, 19 I).*

Animal 13
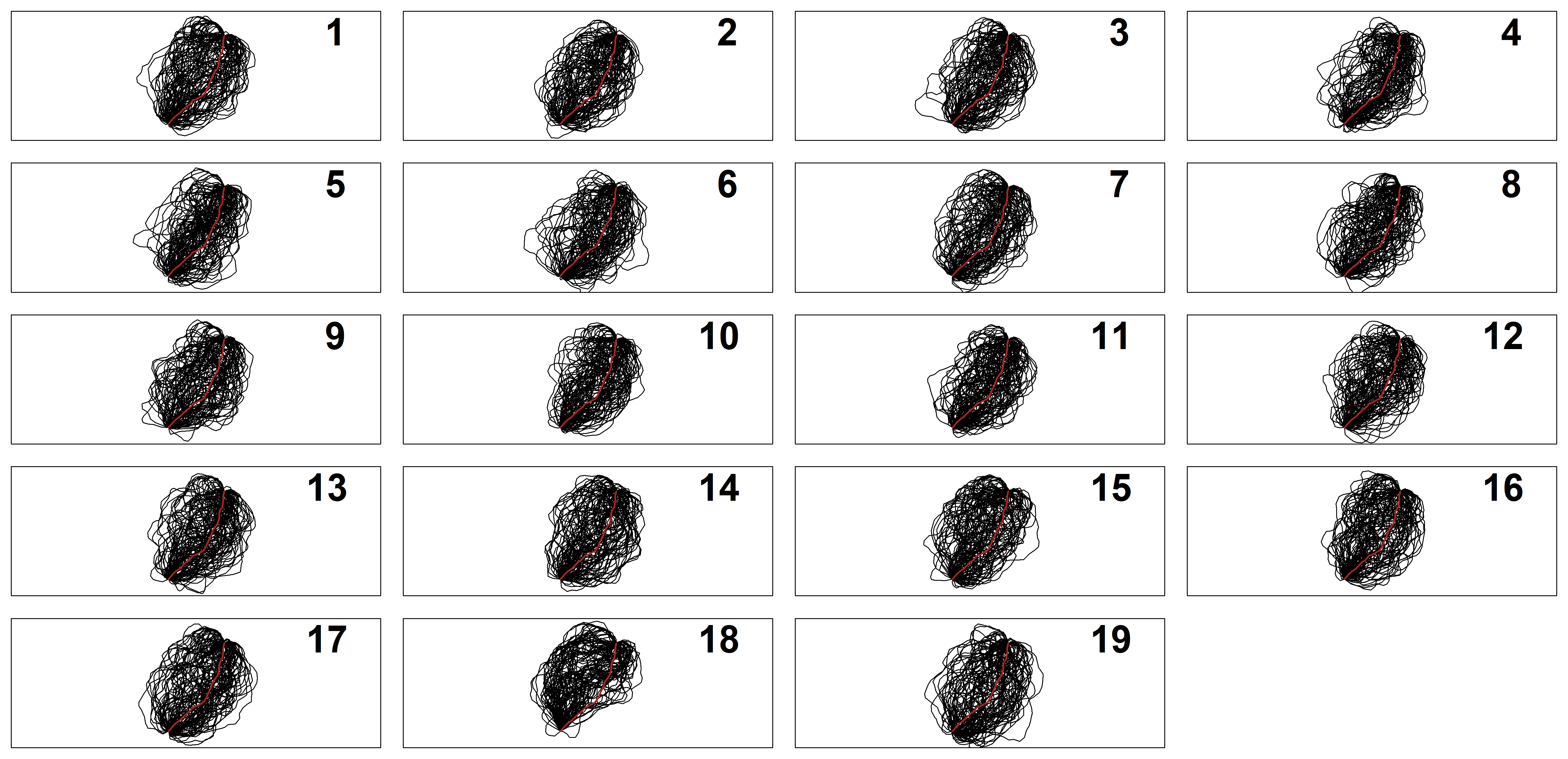


*Figure S29 The sub figures represent the output of the CRB models from 5 navigation strategies with different combinations of geomagnetic inputs (see Table S2 for reference) for animal 13. The labelling is as per the following: No bias (1), Constant heading (2 Max F, 3 Max H, 4 Max I, 5 Min F, 6 Min H, 7 Min I), Combination Bi-gradient taxis-Constant heading (8 FH, 9 FI, 10 HF, 11 HI, 12 IF, 13 IH), Bi-gradient taxis (14 FH, 15 FI, 16 IH), Geomagnetic taxis (17 F, 18 H, 19 I).*

Animal 14
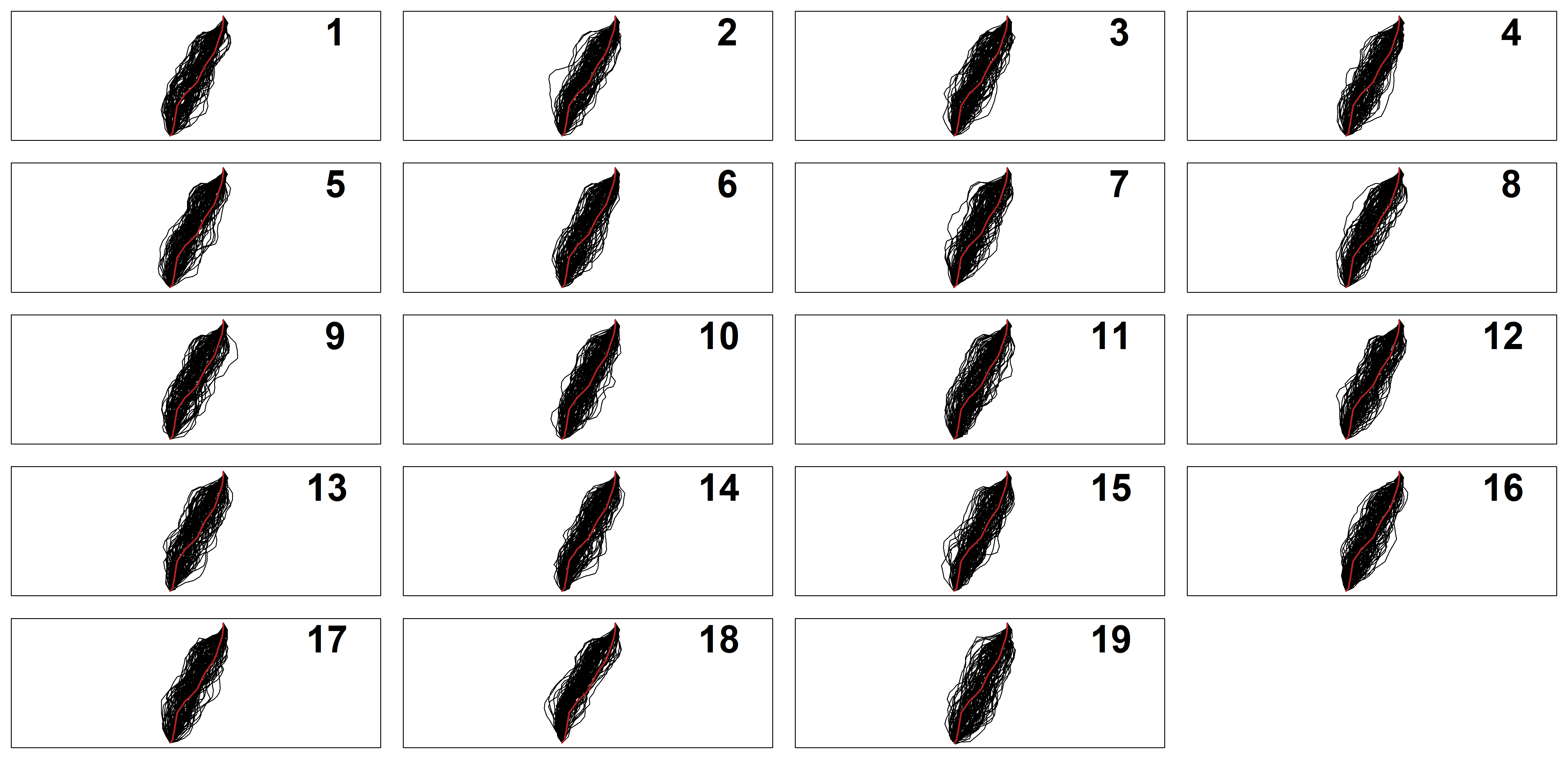


*Figure S30 The sub figures represent the output of the CRB models from 5 navigation strategies with different combinations of geomagnetic inputs (see Table S2 for reference) for animal 14. The labelling is as per the following: No bias (1), Constant heading (2 Max F, 3 Max H, 4 Max I, 5 Min F, 6 Min H, 7 Min I), Combination Bi-gradient taxis-Constant heading (8 FH, 9 FI, 10 HF, 11 HI, 12 IF, 13 IH), Bi-gradient taxis (14 FH, 15 FI, 16 IH), Geomagnetic taxis (17 F, 18 H, 19 I).*

S3 - Statistics

We calculated 6 different linear mixed effect models. For CRW outputs we run 3 different models for the 3 different trajectory similarity measurements (mean distance, mean; dynamic time warping, DTW; dynamic interaction index, DI). These trajectory similarity measurements (independent variable) were modelled separately against the different simulated strategies (dependent variable). To account for autocorrelation, introduced by repeated measures, we added a random intercept effect term for animal identity. These 3 linear mixed effect models were calculated for CRW and CRB separately.

| **CRW** |  |  |  |  |  |  |
| --- | --- | --- | --- | --- | --- | --- |
|  |  |  |  |  |  |  |
| **Model 1 - Mean** |  |  |  |  |  |  |
| lmer(meandis~strategy2+(1\|animalID),data = statsall_crw) | | |  |  |  |  |
| Linear mixed model fit by REML. t-tests use Satterthwaite's method | | | |  |  |  |
| REML criterion at convergence: 748106.5 | |  |  |  |  |  |
|  |  |  |  |  |  |  |
| Scaled residuals: |  |  |  |  |  |  |
| Min | 1Q | Median | 3Q | Max |  |  |
| -3.7395 | -0.7480 | -0.0581 | 0.6954 | 3.7500 |  |  |
|  |  |  |  |  |  |  |
| Random effects: |  |  |  |  |  |  |
| Groups | Name | Variance | Std.Dev. |  |  |  |
| animalID | (Intercept) | 11430353643 | 106913 |  |  |  |
| Residual |  | 97070590101 | 311562 |  |  |  |
| Number of obs: 26600, groups: animalID, 14 | |  |  |  |  |  |
|  |  |  |  |  |  |  |
| Fixed effects: |  |  |  |  |  |  |
|  | Estimate | Std.Error | df | t value | Pr(>\|t\|) |  |
| (Intercept) | 1097460 | 29762 | 15 | 36.87 | <3E-16 | *** |
| strategy_con_F | -313605 | 11776 | 26568 | -26.63 | <2E-16 | *** |
| strategy_con_H | -309741 | 11776 | 26568 | -26.3 | <2E-16 | *** |
| strategy_con_Inc | -319608 | 11776 | 26568 | -27.14 | <2E-16 | *** |
| strategy_conMIN_F | -310657 | 11776 | 26568 | -26.38 | <2E-16 | *** |
| strategy_conMIN_H | -300773 | 11776 | 26568 | -25.54 | <2E-16 | *** |
| strategy_conMIN_Inc | -321866 | 11776 | 26568 | -27.33 | <2E-16 | *** |
| strategy_combined_FH | -338430 | 11776 | 26568 | -28.74 | <2E-16 | *** |
| strategy_combined_FInc | -340320 | 11776 | 26568 | -28.9 | <2E-16 | *** |
| strategy_combined_HF | -329440 | 11776 | 26568 | -27.98 | <2E-16 | *** |
| strategy_combined_HInc | -326552 | 11776 | 26568 | -27.73 | <2E-16 | *** |
| strategy_combined_IncF | -312994 | 11776 | 26568 | -26.58 | <2E-16 | *** |
| strategy_combined_IncH | -320948 | 11776 | 26568 | -27.25 | <2E-16 | *** |
| strategy_bi-grad_FH | -237266 | 11776 | 26568 | -20.15 | <2E-16 | *** |
| strategy_bi-grad_FInc | -250860 | 11776 | 26568 | -21.3 | <2E-16 | *** |
| strategy_bi-grad_IncH | -244449 | 11776 | 26568 | -20.76 | <2E-16 | *** |
| strategy_max_F | -425952 | 11776 | 26568 | -36.17 | <2E-16 | *** |
| strategy_max_H | -47694 | 11776 | 26568 | -4.05 | 5.13E-05 | *** |
| strategy_max_Inc | -398224.85 | 11776 | 26568 | -33.82 | <2E-16 | *** |
| Signif. codes: 0 '***' 0.001 '**' 0.01 '*' 0.05 '.' 0.1 ' ' 1 | | |  |  |  |  |
|  |  |  |  |  |  |  |
| **Model 2 - DTW** |  |  |  |  |  |  |
| lmer(dtw~strategy2+(1\|animalID),data = statsall_crw) | | |  |  |  |  |
| Linear mixed model fit by REML. t-tests use Satterthwaite's method | | | |  |  |  |
| REML criterion at convergence: 965120.3 | |  |  |  |  |  |
|  |  |  |  |  |  |  |
| Scaled residuals: |  |  |  |  |  |  |
| Min | 1Q | Median | 3Q | Max |  |  |
| -3.4580 | -0.6692 | -0.0845 | 0. 5872 | 4.8528 |  |  |
|  |  |  |  |  |  |  |
| Random effects: |  |  |  |  |  |  |
| Groups | Name | Variance | Std.Dev. |  |  |  |
| animalID | (Intercept) | 1.46946E+14 | 12122132 |  |  |  |
| Residual |  | 3.40800E+14 | 18460771 |  |  |  |
| Number of obs: 26600, groups: animalID, 14 | |  |  |  |  |  |
|  |  |  |  |  |  |  |
| Fixed effects: |  |  |  |  |  |  |
|  | Estimate | Std.Error | df | t value | Pr(>\|t\|) |  |
| (Intercept) | 53114042 | 3277129 | 14 | 16 | 2.85E-10 | *** |
| strategy_con_F | -17117654 | 697752 | 1.56E+20 | -24.53 | <2E-16 | *** |
| strategy_con_H | -17045904 | 697752 | 1.46E+20 | -24.43 | <2E-16 | *** |
| strategy_con_Inc | -17533654 | 697752 | 1.47E+20 | -25.13 | <2E-16 | *** |
| strategy_conMIN_F | -16830473 | 697752 | 1.51E+20 | -24.12 | <2E-16 | *** |
| strategy_conMIN_H | -16826717 | 697752 | 1.50E+20 | -24.12 | <2E-16 | *** |
| strategy_conMIN_Inc | -17988945 | 697752 | 1.50E+20 | -25.78 | <2E-16 | *** |
| strategy_combined_FH | -18981289 | 697752 | 1.50E+20 | -27.20 | <2E-16 | *** |
| strategy_combined_FInc | -19165204 | 697752 | 1.57E+20 | -27.47 | <2E-16 | *** |
| strategy_combined_HF | -18089721 | 697752 | 1.58E+20 | -25.93 | <2E-16 | *** |
| strategy_combined_HInc | -17933042 | 697752 | 1.51E+20 | -25.70 | <2E-16 | *** |
| strategy_combined_IncF | -17748879 | 697752 | 1.59E+20 | -25.44 | <2E-16 | *** |
| strategy_combined_IncH | -18086219 | 697752 | 1.50E+20 | -25.92 | <2E-16 | *** |
| strategy_bi-grad_FH | -13162168 | 697752 | 1.32E+20 | -18.86 | <2E-16 | *** |
| strategy_bi-grad_FInc | -13794590 | 697752 | 1.46E+20 | -19.77 | <2E-16 | *** |
| strategy_bi-grad_IncH | -13370639 | 697752 | 1.54E+20 | -19.16 | <2E-16 | *** |
| strategy_max_F | -22903128 | 697752 | 1.51E+20 | -32.82 | <2E-16 | *** |
| strategy_max_H | -1458142 | 697752 | 1.38E+20 | -2.09 | 0.0366 | * |
| strategy_max_Inc | -21294469 | 697752 | 1.62E+20 | -30.52 | <2E-16 | *** |
| Signif. codes: 0 '***' 0.001 '**' 0.01 '*' 0.05 '.' 0.1 ' ' 1 | | |  |  |  |  |
|  |  |  |  |  |  |  |
| **Model 3 - DI index** |  |  |  |  |  |  |
| lmer(sim1~strategy2+(1\|animalID),data = statsall_crw) | | |  |  |  |  |
| Linear mixed model fit by REML. t-tests use Satterthwaite's method | | | |  |  |  |
| REML criterion at convergence: 6166.9 | |  |  |  |  |  |
|  |  |  |  |  |  |  |
| Scaled residuals: |  |  |  |  |  |  |
| Min | 1Q | Median | 3Q | Max |  |  |
| -3.5525 | -0.6973 | 0. 0351 | 0. 7466 | 2.8382 |  |  |
|  |  |  |  |  |  |  |
| Random effects: |  |  |  |  |  |  |
| Groups | Name | Variance | Std.Dev. |  |  |  |
| animalID | (Intercept) | 0.00100 | 0.03162 |  |  |  |
| Residual |  | 0.07334 | 0.27081 |  |  |  |
| Number of obs: 26600, groups: animalID, 14 | |  |  |  |  |  |
|  |  |  |  |  |  |  |
| Fixed effects: |  |  |  |  |  |  |
|  | Estimate | Std.Error | df | t value | Pr(>\|t\|) |  |
| (Intercept) | 0.011400 | 0.011130 | 36 | 1.03 | 0.312 |  |
| strategy_con_F | 0.181600 | 0.010240 | 26568 | 17.74 | <2E-16 | *** |
| strategy_con_H | 0.175600 | 0.010240 | 26568 | 17.16 | <2E-16 | *** |
| strategy_con_Inc | 0.185030 | 0.010240 | 26568 | 18.08 | <2E-16 | *** |
| strategy_conMIN_F | 0.192290 | 0.010240 | 26568 | 18.79 | <2E-16 | *** |
| strategy_conMIN_H | 0.179880 | 0.010240 | 26568 | 17.57 | <2E-16 | *** |
| strategy_conMIN_Inc | 0.188760 | 0.010240 | 26568 | 18.44 | <2E-16 | *** |
| strategy_combined_FH | 0.208230 | 0.010240 | 26568 | 20.34 | <2E-16 | *** |
| strategy_combined_FInc | 0.203700 | 0.010240 | 26568 | 19.90 | <2E-16 | *** |
| strategy_combined_HF | 0.193000 | 0.010240 | 26568 | 18.86 | <2E-16 | *** |
| strategy_combined_HInc | 0.182680 | 0.010240 | 26568 | 17.85 | <2E-16 | *** |
| strategy_combined_IncF | 0.180780 | 0.010240 | 26568 | 17.66 | <2E-16 | *** |
| strategy_combined_IncH | 0.187840 | 0.010240 | 26568 | 18.35 | <2E-16 | *** |
| strategy_bi-grad_FH | 0.111050 | 0.010240 | 26568 | 10.85 | <2E-16 | *** |
| strategy_bi-grad_FInc | 0.122680 | 0.010240 | 26568 | 11.99 | <2E-16 | *** |
| strategy_bi-grad_IncH | 0.115270 | 0.010240 | 26568 | 11.26 | <2E-16 | *** |
| strategy_max_F | 0.310920 | 0.010240 | 26568 | 30.38 | <2E-16 | *** |
| strategy_max_H | -0.049310 | 0.010240 | 26568 | -4.82 | <1.5E-06 | *** |
| strategy_max_Inc | 0.277840 | 0.010240 | 26568 | 27.14 | <2E-16 | *** |
| Signif. codes: 0 '***' 0.001 '**' 0.01 '*' 0.05 '.' 0.1 ' ' 1 | | |  |  |  |  |
|  |  |  |  |  |  |  |
|  |  |  |  |  |  |  |
| **CRB** |  |  |  |  |  |  |
| **Model 4 - Mean** |  |  |  |  |  |  |
| lmer(meandis~strategy2+(1\|animalID),data = statsall_bcrw) | | |  |  |  |  |
| Linear mixed model fit by REML. t-tests use Satterthwaite's method [ | | | |  |  |  |
| REML criterion at convergence: 664788.3 | |  |  |  |  |  |
|  |  |  |  |  |  |  |
| Scaled residuals: |  |  |  |  |  |  |
| Min | 1Q | Median | 3Q | Max |  |  |
| -5.1987 | -0.5467 | -0.0537 | 0.4395 | 5.8487 |  |  |
|  |  |  |  |  |  |  |
| Random effects: |  |  |  |  |  |  |
| Groups | Name | Variance | Std.Dev. |  |  |  |
| animalID | (Intercept) | 8905556103 | 94369 |  |  |  |
| Residual |  | 4218694908 | 64951 |  |  |  |
| Number of obs: 26600, groups: animalID, 14 | |  |  |  |  |  |
|  |  |  |  |  |  |  |
| Fixed effects: |  |  |  |  |  |  |
|  | Estimate | Std.Error | df | t value | Pr(>\|t\|) |  |
| (Intercept) | 229201.6 | 25280.91 | 13.12 | 9.07 | 5.17E-07 | *** |
| strategy2con_F | -8638.4 | 2454.94 | 26568 | -3.52 | 4.34E-04 | *** |
| strategy2con_H | -10653.8 | 2454.94 | 26568 | -4.34 | 1.43E-05 | *** |
| strategy2con_Inc | -13891.8 | 2454.94 | 26568 | -5.66 | 1.54E-08 | *** |
| strategy2conMIN_F | -15079.6 | 2454.94 | 26568 | -6.14 | 8.24E-10 | *** |
| strategy2conMIN_H | -10899.6 | 2454.94 | 26568 | -4.44 | 9.04E-06 | *** |
| strategy2conMIN_Inc | -12800.2 | 2454.94 | 26568 | -5.21 | 1.86E-07 | *** |
| strategy2combined_FH | -12443.6 | 2454.94 | 26568 | -5.07 | 4.03E-07 | *** |
| strategy2combined_FInc | -11449.6 | 2454.94 | 26568 | -4.66 | 3.12E-06 | *** |
| strategy2combined_HF | -14611.7 | 2454.94 | 26568 | -5.95 | 2.68E-09 | *** |
| strategy2combined_HInc | -13444.4 | 2454.94 | 26568 | -5.48 | 4.38E-08 | *** |
| strategy2combined_IncF | -10730.8 | 2454.94 | 26568 | -4.37 | 1.24E-05 | *** |
| strategy2combined_IncH | -13667.5 | 2454.94 | 26568 | -5.57 | 2.61E-08 | *** |
| strategy2bi-grad_FH | -1884.2 | 2454.94 | 26568 | -0.77 | 4.43E-01 |  |
| strategy2bi-grad_FInc | -2711.7 | 2454.94 | 26568 | -1.11 | 2.69E-01 |  |
| strategy2bi-grad_IncH | -3707.7 | 2454.94 | 26568 | -1.51 | 1.31E-01 |  |
| strategy2max_F | -8998.2 | 2454.94 | 26568 | -3.67 | 2.48E-04 | *** |
| strategy2max_H | 40292.5 | 2454.94 | 26568 | 16.41 | <2E-16 | *** |
| strategy2max_Inc | -8455.1 | 2454.94 | 26568 | -3.44 | 5.74E-04 | *** |
| Signif. codes: 0 '***' 0.001 '**' 0.01 '*' 0.05 '.' 0.1 ' ' 1 | | |  |  |  |  |
|  |  |  |  |  |  |  |
| **Model 5 - DTW** |  |  |  |  |  |  |
| lmer(dtw~strategy2+(1\|animalID),data = statsall_bcrw) | | |  |  |  |  |
| Linear mixed model fit by REML. t-tests use Satterthwaite's method [ | | | |  |  |  |
| REML criterion at convergence: 889890.5 | |  |  |  |  |  |
|  |  |  |  |  |  |  |
| Scaled residuals: |  |  |  |  |  |  |
| Min | 1Q | Median | 3Q | Max |  |  |
| -3.9141 | -0.5066 | -0.1088 | 0.3609 | 6.4602 |  |  |
|  |  |  |  |  |  |  |
| Random effects: |  |  |  |  |  |  |
| Groups | Name | Variance | Std.Dev. |  |  |  |
| animalID | (Intercept) | 2.86287E+13 | 5350580 |  |  |  |
| Residual |  | 2.00956E+13 | 4482816 |  |  |  |
| Number of obs: 26600, groups: animalID, 14 | |  |  |  |  |  |
|  |  |  |  |  |  |  |
| Fixed effects: |  |  |  |  |  |  |
|  | Estimate | Std.Error | df | t value | Pr(>\|t\|) |  |
| (Intercept) | 9467800.5 | 1435012.88 | 13.17 | 6.60 | 1.61E-05 | *** |
| strategy2con_F | -558981.9 | 169434.54 | 2.02761E+21 | -3.30 | 9.70E-04 | *** |
| strategy2con_H | -814943.5 | 169434.54 | 2.09193E+21 | -4.81 | 1.51E-06 | *** |
| strategy2con_Inc | -776807.7 | 169434.54 | 1.84796E+21 | -4.59 | 4.55E-06 | *** |
| strategy2conMIN_F | -1035532.1 | 169434.54 | 2.20168E+21 | -6.11 | 9.86E-10 | *** |
| strategy2conMIN_H | -658652.2 | 169434.54 | 1.76739E+21 | -3.89 | 1.01E-04 | *** |
| strategy2conMIN_Inc | -886027.5 | 169434.54 | 1.73691E+21 | -5.23 | 1.70E-07 | *** |
| strategy2combined_FH | -763414.5 | 169434.54 | 1.73854E+21 | -4.51 | 6.62E-06 | *** |
| strategy2combined_FInc | -803780.6 | 169434.54 | 2.09193E+21 | -4.74 | 2.10E-06 | *** |
| strategy2combined_HF | -972455.9 | 169434.54 | 2.67061E+21 | -5.74 | 9.50E-09 | *** |
| strategy2combined_HInc | -814082.3 | 169434.54 | 1.90395E+21 | -4.81 | 1.55E-06 | *** |
| strategy2combined_IncF | -724809.9 | 169434.54 | 2.08671E+21 | -4.28 | 1.89E-05 | *** |
| strategy2combined_IncH | -855262.7 | 169434.54 | 2.1312E+21 | -5.05 | 4.47E-07 | *** |
| strategy2bi-grad_FH | 27412.7 | 169434.54 | 1.21008E+21 | 0.16 | 8.71E-01 |  |
| strategy2bi-grad_FInc | 22969.8 | 169434.54 | 1.57109E+21 | 0.14 | 8.92E-01 |  |
| strategy2bi-grad_IncH | -265772.4 | 169434.54 | 1.78784E+21 | -1.57 | 1.17E-01 |  |
| strategy2max_F | 258064.5 | 169434.54 | 1.35314E+21 | 1.52 | 1.28E-01 |  |
| strategy2max_H | 2094982.1 | 169434.54 | 1.99609E+21 | 12.37 | <2E-16 | *** |
| strategy2max_Inc | 249978.9 | 169434.54 | 1.84537E+21 | 1.48 | 1.40E-01 |  |
| Signif. codes: 0 '***' 0.001 '**' 0.01 '*' 0.05 '.' 0.1 ' ' 1 | | |  |  |  |  |
|  |  |  |  |  |  |  |
| **Model 6 - DI index** |  |  |  |  |  |  |
| lmer(sim1~strategy2+(1\|animalID),data = statsall_bcrw) | | |  |  |  |  |
| Linear mixed model fit by REML. t-tests use Satterthwaite's method | | | |  |  |  |
| REML criterion at convergence: -77523.7 | |  |  |  |  |  |
|  |  |  |  |  |  |  |
| Scaled residuals: |  |  |  |  |  |  |
| Min | 1Q | Median | 3Q | Max |  |  |
| -5.3846 | -0.562 | 0.0282 | 0.5772 | 5.2213 |  |  |
|  |  |  |  |  |  |  |
| Random effects: |  |  |  |  |  |  |
| Groups | Name | Variance | Std.Dev. |  |  |  |
| animalID | (Intercept) | 0.021155 | 0.14545 |  |  |  |
| Residual |  | 0.003138 | 0.05602 |  |  |  |
| Number of obs: 26600, groups: animalID, 14 | |  |  |  |  |  |
|  |  |  |  |  |  |  |
| Fixed effects: |  |  |  |  |  |  |
|  | Estimate | Std.Error | df | t value | Pr(>\|t\|) |  |
| (Intercept) | 0.6396595 | 0.0389017 | 13.0360968 | 16.44 | 4.25E-10 | *** |
| strategy2con_F | 0.0001387 | 0.0021173 | 26568 | 0.07 | 9.48E-01 |  |
| strategy2con_H | 0.0032694 | 0.0021173 | 26568 | 1.54 | 1.23E-01 |  |
| strategy2con_Inc | 0.006874 | 0.0021173 | 26568 | 3.25 | 1.17E-03 | ** |
| strategy2conMIN_F | 0.002482 | 0.0021173 | 26568 | 1.17 | 2.41E-01 |  |
| strategy2conMIN_H | 0.0050066 | 0.0021173 | 26568 | 2.37 | 1.81E-02 | * |
| strategy2conMIN_Inc | 0.00238 | 0.0021173 | 26568 | 1.12 | 2.61E-01 |  |
| strategy2combined_FH | 0.0042078 | 0.0021173 | 26568 | 1.99 | 4.69E-02 | * |
| strategy2combined_FInc | 0.0025519 | 0.0021173 | 26568 | 1.21 | 2.28E-01 |  |
| strategy2combined_HF | 0.0058688 | 0.0021173 | 26568 | 2.77 | 5.58E-03 | ** |
| strategy2combined_HInc | 0.0028073 | 0.0021173 | 26568 | 1.33 | 1.85E-01 |  |
| strategy2combined_IncF | 0.0041257 | 0.0021173 | 26568 | 1.95 | 5.14E-02 | . |
| strategy2combined_IncH | 0.0054148 | 0.0021173 | 26568 | 2.56 | 1.06E-02 | * |
| strategy2bi-grad_FH | -0.0017988 | 0.0021173 | 26568 | -0.85 | 3.96E-01 |  |
| strategy2bi-grad_FInc | -0.0017421 | 0.0021173 | 26568 | -0.82 | 4.11E-01 |  |
| strategy2bi-grad_IncH | 0.0025146 | 0.0021173 | 26567.99999 | 1.19 | 2.35E-01 |  |
| strategy2max_F | 0.0041994 | 0.0021173 | 26568 | 1.98 | 4.73E-02 | * |
| strategy2max_H | -0.014005 | 0.0021173 | 26568 | -6.62 | 3.79E-11 | *** |
| strategy2max_Inc | 0.0062692 | 0.0021173 | 26568 | 2.96 | 3.07E-03 | ** |
| Signif. codes: 0 '***' 0.001 '**' 0.01 '*' 0.05 '.' 0.1 ' ' 1 | | |  |  |  |  |
